# SIRT6‐Mediated Deacetylation of ATF3 Promotes Silica‐Induced Lung Fibrosis by Enhancing its Nuclear Import via Binding to Importin α

**DOI:** 10.1002/advs.75782

**Published:** 2026-05-20

**Authors:** Demin Cheng, Wenxia Bu, Fengxu Wang, Yueyuan Jin, Rongzhu Liu, Rui Zhao, Xuehai Wang, Mengna Jiang, Jinping Shen, Xinhang Cheng, Zuming Chen, Li Zhu, Jinlong Li, Zhenzhong Ge, Shichen Miao, Haotian Xu, Xiaoyu Zhou, Dongming Wang, Xinyuan Zhao

**Affiliations:** ^1^ Department of Respiratory and Critical Care Medicine Affiliated Hospital of Nantong University School of Public Health Nantong University Nantong China; ^2^ Department of Respiratory Wuxi No 8 People's Hospital Wuxi Jiangsu China; ^3^ School of Public Health North China University of Science and Technology Tangshan Hebei China; ^4^ Department of Breast Surgery Shanghai Key Laboratory of Maternal Fetal Medicine Shanghai Institute of Maternal‐Fetal Medicine and Gynecologic Oncology Shanghai First Maternity and Infant Hospital School of Medicine Tongji University Shanghai China; ^5^ Department of Occupational & Environmental Health School of Public Health Tongji Medical College Huazhong University of Science and Technology Wuhan Hubei China; ^6^ Key Laboratory of Environment and Health Ministry of Education & Ministry of Environmental Protection and State Key Laboratory of Environmental Health (Incubating) School of Public Health Tongji Medical College Huazhong University of Science and Technology Wuhan Hubei China

**Keywords:** ATF3 deacetylation, cellular senescence, nuclear entry, pulmonary fibrosis, silicosis

## Abstract

Silicosis is the most common occupational lung disease caused by respirable crystalline silica inhalation, with limited therapeutic options. Cellular senescence plays a critical role in the pathogenesis of lung diseases, while the role of senescent macrophages in silicosis remains unclear. Single‐cell RNA sequencing (scRNA‐seq) of healthy and silicosis human and mouse lung tissues revealed that activating transcription factor 3 (ATF3)‐mediated macrophage senescence is closely linked to silicosis progression. Mechanistically, Sirtuin 6 (SIRT6)‐mediated ATF3 deacetylation enhanced its nuclear transport and subsequently activated mitochondria‐localized glutamic acid‐rich protein (MGARP) transcription, thereby causing mitochondrial dysfunction and macrophage senescence. Senescent macrophages promoted fibroblast activation via the secreted phosphoprotein 1 (SPP1)‐cluster of differentiation 44 (CD44) signaling pathway. Furthermore, the nuclear transport protein importin α and the molecular chaperone protein heat shock protein 70 (HSP70) competitively bound to ATF3, preventing its lysosomal degradation while promoting its nuclear import during macrophage senescence. Moreover, the small‐molecule inhibitor Itraconazole, which targets the binding site of ATF3 and importin α, could reduce ATF3 nuclear entry, macrophage senescence, and pulmonary fibrosis (PF). Collectively, our study provided insights into the mechanism by which deacetylated ATF3 facilitates silicosis progression via increased nuclear transport and macrophage senescence, and indicated potential therapeutic targets for PF.

## Graphical abstract

SIRT6‐mediated ATF3 acetylation drives MGARP transcription and mitochondrial dysfunction in macrophages, promoting macrophage senescence and pulmonary fibrosis. Mechanistically, HSP70/Importin α competitively binds to ATF3, modulating its nuclear translocation. Importantly, targeting ATF3‐Importin α interaction alleviates silica‐induced pulmonary fibrosis, revealing new insights into macrophage senescence in silicosis.

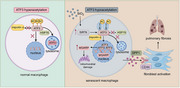

## 1 Introduction

Silicosis is an ancient and fatal occupational lung disease caused by the prolonged exposure to respirable crystalline silica, characterized by inflammatory cascades, destruction of alveolar structure, excessive deposition of extracellular matrix (ECM), and eventually respiratory failure [1, 2]. While mining and traditional stone processing represent major sources of silica exposure, emerging sources like denim sandblasting and artificial stone benchtops manufacturing have raised the prevalence of silicosis, both in developed and developing countries [3, 4]. Despite past extensive studies, the mechanisms underlying silicosis pathology are still poorly understood [5, 6, 7]. Currently, apart from lung transplantation, no other effective treatments are available to treat silicosis. Therefore, it is urgent to provide potential targets and strategies for the clinical treatment of silicosis.

Cellular senescence represents a state of irreversible cell cycle arrest characterized by the robust secretion of diverse bioactive factors, collectively termed the senescence‐associated secretory phenotype (SASP) [8, 9]. Multiple factors can trigger cellular senescence, including mitochondrial damage, telomere shortening, DNA damage, and oncogene activation [10, 11]. Pathologically, persistent cellular senescence releases SASP into the tissue microenvironment and is linked to chronic tissue damage and inflammation, which leads to abnormal tissue remodeling and organ fibrosis [12, 13]. The elimination of senescent cells has been shown to effectively mitigate various pulmonary interstitial diseases, including idiopathic pulmonary fibrosis (IPF) and silicosis [14, 15]. Consequently, it is urgently needed to explore the exact molecular mechanism of cellular senescence during the progression of silicosis, providing a way for developing novel therapeutic strategies.

Macrophages are innate immune cells that are essential for fighting infections, tissue repair, orchestrating immune responses, and maintaining tissue homeostasis [16]. Alveolar macrophages engulf silica particles and secret pro‐fibrotic cytokines, which stimulate fibroblast proliferation, ECM deposition, and fibrosis [2]. As macrophages undergo a state of senescence, they produce elevated levels of inflammatory cytokines and reactive oxygen species (ROS) and exhibit diminished phagocytic capacity, which contribute to inflammation and affect tissue homeostasis [17, 18].

The large variety of epigenetic alterations, including aberrant chromatin remodeling, DNA methylation, histone and non‐histone protein acetylation, plays a crucial role in aging and cellular senescence [19]. Here, we demonstrated that SIRT6‐mediated ATF3 deacetylation promoted ATF3 nuclear translocation, which increased MGARP transcription, mitochondrial damage, and macrophage senescence during silicosis. Senescent macrophage induced fibroblast activation, contraction and migration through the SPP1‐CD44 pathway. Furthermore, we showed that a small‐molecule inhibitor of ATF3 nuclear translocation could attenuate mitochondrial damage and macrophage senescence. Collectively, our study provided insights into the mechanism by which deacetylated ATF3 facilitates silicosis progression via increased nuclear transport and macrophage senescence, and indicated potential therapeutic targets for silica‐induced PF.

## 2 Results

### 2.1 Increased Macrophage Senescence in Silicosis Human and Murine Lung Tissues

**FIGURE 1:**
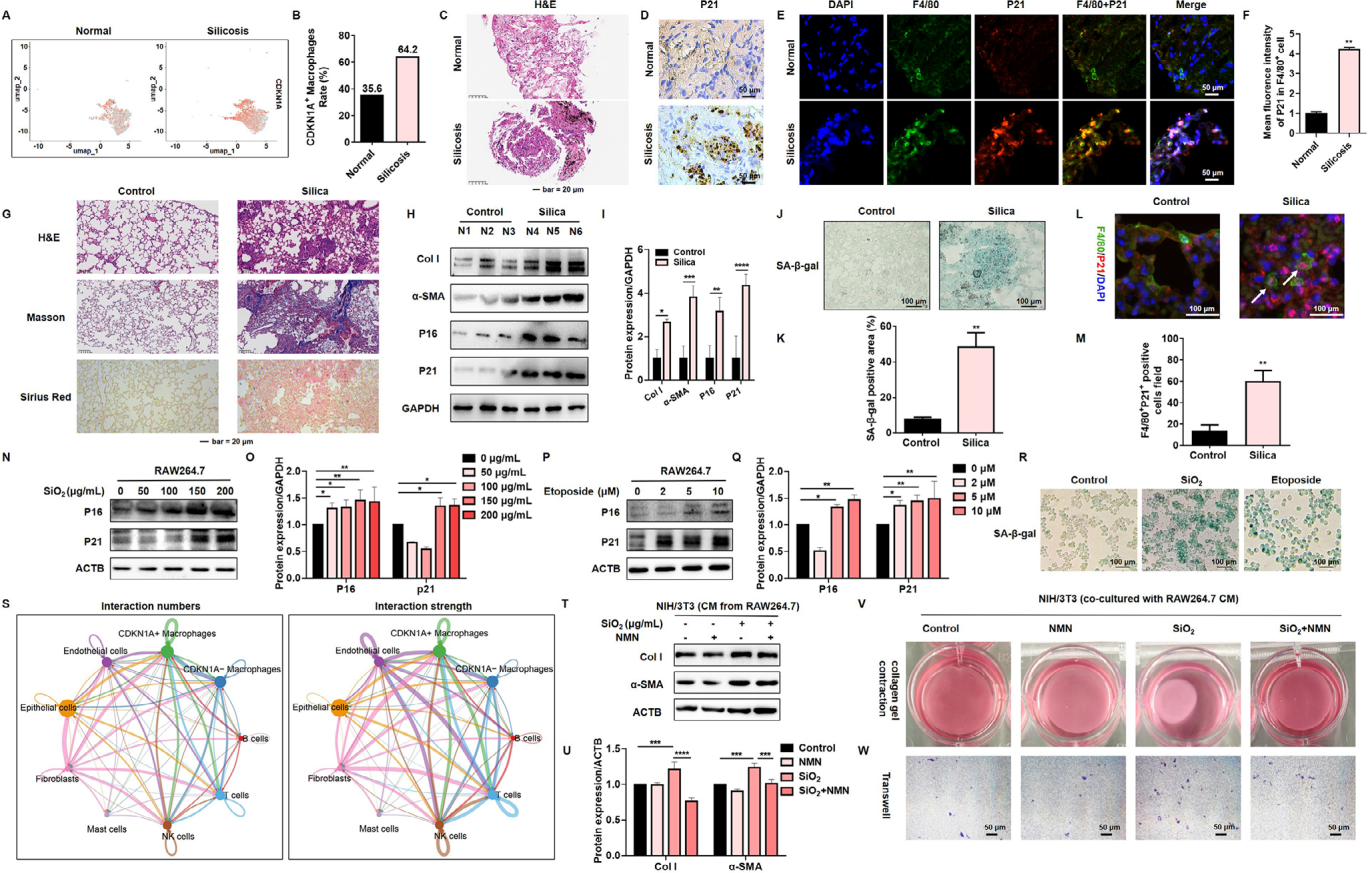
Increased macrophage senescence in silicosis human and murine lung tissues. (A) Uniform manifold approximation and projection (UMAP) plots show CDKN1A‐positive macrophages of normal and silica‐induced fibrotic human lungs (n = 3 for each group, biological replicates). (B) Bar graph representing the rate of CDKN1A‐positive macrophages in the normal and silicosis groups. (C) Typical images of hematoxylin and eosin (H&E) staining of human lung sections from the normal and silicosis patient lung tissues (n = 3 for each group, biological replicates); scale bar = 20 µm. Each experiment was performed in triplicate to ensure reproducibility of results. (D) Typical images of P21 immunohistochemistry (IHC) staining in normal and silicosis patient lung tissues, scale bar = 20 µm. Each experiment was performed in triplicate to ensure reproducibility of results. (E) Representative results for coimmunostaining of F4/80 and P21 in human lung sections from the normal and silicosis groups. Green represents F4/80, red represents P21, and blue represents nuclear DNA staining by blue represents nuclear DNA staining by 4’,6‐diamidino‐2‐phenylindole (DAPI), scale bar = 50 µm. Each experiment was performed in triplicate to ensure reproducibility of results. (F) Mean fluorescence intensity of P21 in F4/80‐positive cells from the lung sections of normal and silicosis groups, all data were expressed as the means ± SD of at least 3 independent experiments, with ^**^ *p* < 0.01 vs the normal group, and p values were from a 2‐tailed unpaired Student's *t*‐test. (G) Representative histopathological images of H&E, Masson, and Sirius red staining of mouse lung tissues from the control and silica groups (n = 3 for each group, biological replicates); scale bar = 20 µm. Each experiment was performed in triplicate to ensure reproducibility of results. (H, I) Western blot and quantitative analysis of fibrotic markers (Col I and α‐SMA) and cellular senescence markers (P16 and P21) in mouse lung tissues from the control and silica groups, all data were expressed as the means ± SD of at least 3 independent experiments, with ^*^ *p* < 0.05, ^**^ *p* < 0.01, ^***^ *p* < 0.001, and ^****^ *p* < 0.0001 vs the indicated group and p values were from a 1‐way ANOVA post–hoc test with Tukey's correction. (J, K) SA‐β‐gal staining and positive area rate in mouse lung tissues from the control and silica groups, all data were expressed as the means ± SD of at least 3 independent experiments, with ^**^ *p* < 0.01 vs the indicated group, and *p* values were from a 2‐tailed unpaired Student's t‐test. (L, M) Co‐immunostaining of F4/80 and P21 and the rate of F4/80^+^ P21^+^ positive cells in the field of mouse lung sections from the control and silica groups, all data were expressed as the means ± SD of at least 3 independent experiments, with ^**^ *p* < 0.01 vs the indicated group, and p values were from a 2‐tailed unpaired Student's *t*‐test. (N, O) Western blot and quantitative analysis of cellular senescence markers (P16 and P21) in SiO_2_‐treated RAW264.7 macrophages, all data were expressed as the means ± SD of at least 3 independent experiments, with ^*^ *p* < 0.05 and ^**^ *p* < 0.01 vs the indicated group, and p values were from a 1‐way ANOVA post–hoc test with Tukey's correction. (P, Q) Western blot and quantitative analysis of cellular senescence markers (P16 and P21) in Etoposide‐treated RAW264.7 macrophages, all data were expressed as the means ± SD of at least 3 independent experiments, with ^*^ *p* < 0.05 and ^**^ *p* < 0.01 vs the indicated group, and *p* values were from a 1‐way ANOVA post–hoc test with Tukey's correction. (R) Representative images of SA‐β‐gal staining in RAW264.7 macrophages from control, SiO_2,_ and Etoposide‐treated groups, scale bar = 100 µm. Each experiment was performed in triplicate to ensure reproducibility of results. (S) Interactions numbers and strength between the different subpopulations from scRNA‐seq data of normal and silicotic human lung tissues. (T, U) Western blot and quantitative analysis of fibrotic markers (Col I and α‐SMA) in NIH/3T3 cells, all data were expressed as the means ± SD of at least 3 independent experiments, with ^***^ *p* < 0.001 and ^****^ *p* < 0.0001 vs the indicated group, and *p* values were from a 1‐way ANOVA post–hoc test with Tukey's correction. (V) Fibroblast contraction was measured using the collagen gel‐based contraction assay. Each experiment was performed in triplicate to ensure reproducibility of results. (W) Representative micrographs of cell migration in the transwell migration assay. Each experiment was performed in triplicate to ensure reproducibility of results.

To investigate the potential role of cellular senescence in silicosis, we analyzed bulk RNA sequencing (RNA‐seq) data from phosphate‐buffered saline (PBS) and silica‐exposed mice lung tissues. Transcriptomic results indicated that senescence‐related genes were markedly increased (Figure S1A). We further mined the publicly available datasets of scRNA‐seq data generated by samples from lung tissues of three silicosis patients and three healthy donors, revealing a significant increase in macrophage senescence in silicosis patients, which most prominently correlated with enhanced fibroblast crosstalk (Figure 1A,B and Figure S1B–D). Consistently, we observed elevated expression of cyclin‐dependent kinase inhibitor 1A (gene name: CDKN1A; protein name: P21) in human silicotic lung tissues of macrophages (Figure 1C–F). Also, our previous scRNA‐seq data of mouse lung tissues showed that Cdkn1a expression was increased in silica‐treated macrophages (Figure S1E,F). Therefore, our main goal was to analyze the effect of macrophage senescence in silicosis. Next, we utilized a single intratracheal instillation of silica suspension to establish a mouse silicosis model. Representative histopathological analyses, western blot results, organ coefficient of the lung, and hydroxyproline assay showed that silica‐induced PF in mice (Figure 1G–I and Figure S1G–J). At the same time, we found that silica exposure induced cellular senescence in both mouse lung tissues and macrophage cells (Figure 1J–M and Figure S1K).

In vitro, SiO_2_ and etoposide‐treated RAW 264.7 mouse macrophage cells showed a dose‐dependent increase of senescence marker protein P21 and cyclin‐dependent kinase inhibitor 2A (gene name: CDKN2A; protein name: P16) (Figure 1N–Q). Senescence‐associated β‐galactosidase (SA‐β‐gal) staining also revealed a significant indication of cellular senescence after SiO_2_ and etoposide exposure (Figure 1R). Previous studies have shown that macrophage senescence is accompanied by downregulated phagocytosis and altered polarization [20]. Correspondingly, in the cell senescence model of SiO_2_ and etoposide, the macrophages showed diminished capacity to take up the fluorescent microspheres (Figure S1L). However, we did not observe the significant changes in macrophage polarization (Figure S1M–P). Nicotinamide mononucleotide (NMN), a main precursor of NAD+, the supplementation of NMN can effectively alleviate cellular senescence [21]. As expected, treatment with NMN decreased the protein level of P21 and P16, as well as SA‐β‐gal activity in SiO_2_‐exposed macrophages (Figure S1Q–T). Next, we investigated whether senescent macrophages played a pro‐fibrotic or anti‐fibrotic role in the process of silicosis. ScRNA‐seq data of normal and silicotic human lung tissues analysis showed a stronger interaction between CDKN1A^+^ macrophages and fibroblasts (Figure 1S). This finding was also evident from our in vitro experiments, which confirmed the elevated protein of fibrotic markers, contraction, and migration capability in senescent macrophages derived supernatant treated‐fibroblasts (Figure 1T–W). Together, these results indicate that macrophage senescence is increased in silicotic lung tissues and may contribute to promoting fibrogenesis in silicosis.

### 2.2 ATF3 is up‐Regulated in Senescent Macrophages of Human and Murine Silicotic Lungs

**FIGURE 2:**
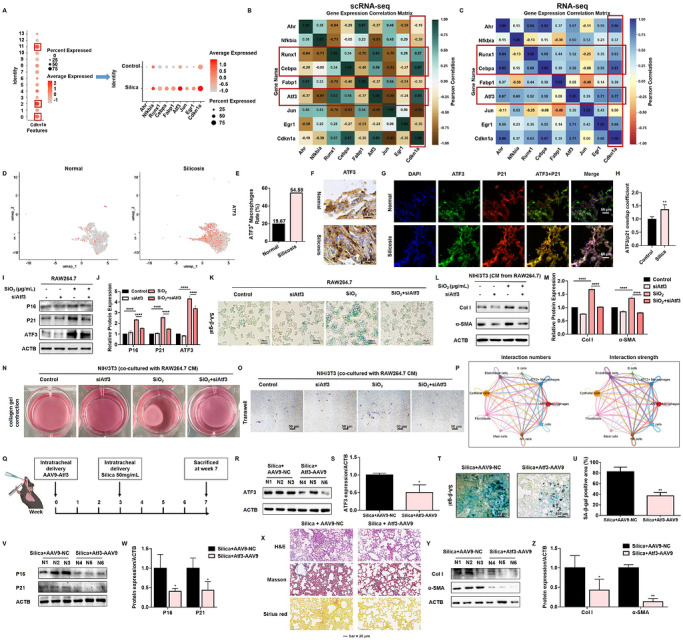
ATF3 is up‐regulated in senescent macrophages of human and murine silicotic lungs. (A) Bubble plot of the top differentially expressed genes (including Ahr, Nfkbia, Runx1, Cebpa, Fabp1, Atf3, Jun, and Egr1) for each macrophage cell cluster from the scRNA‐seq of control and silica‐treated mice (n = 2 for each group, biological replicates). (B) Correlation between the expression of Cdkn1a and differentially expressed genes of macrophage subsets according to scRNA‐seq data from mouse lung tissues of control mice and those exposed to silica for 28 days (n = 2 for each group, biological replicates). (C) Correlation between the expression of Cdkn1a and differentially expressed genes of macrophage subsets according to bulk RNA‐seq data from mouse lung tissues of control mice and those exposed to silica for 28 days (n = 3 for each group, biological replicates). (D) UMAP plots show ATF3‐positive macrophages according to scRNA‐seq data from normal and silica‐induced fibrotic human lungs (n = 3 for each group, biological replicates). (E) Bar graph representing the rate of ATF3‐positive macrophages in normal and silica‐induced fibrotic human lungs. (F) Typical images of ATF3 IHC staining in normal and silicosis patient lung tissues, scale bar = 50 µm. Each experiment was performed in triplicate to ensure reproducibility of results. (G) Representative results for coimmunostaining of P21 and ATF3 in human lung sections from the normal and silicosis groups. Green represents ATF3, red represents P21, and blue represents nuclear DNA staining by DAPI, scale bar = 50 µm. Each experiment was performed in triplicate to ensure reproducibility of results. (H) ATF3/P21 overlap coefficient from the lung sections of normal and silicosis groups, all data were expressed as the means ± SD of at least 3 independent experiments, with ^**^ *p* < 0.01 vs. the indicated group and p values were from a 2‐tailed unpaired Student's *t*‐test. (I,J) Western blot and quantitative analysis of cellular senescence markers (P16 and P21) and ATF3 in RAW264.7 macrophages, all data were expressed as the means ± SD of at least 3 independent experiments, with ^****^ *p* < 0.0001 vs. the indicated group, and *p* values were from a 1‐way ANOVA post–hoc test with Tukey's correction. (K) Representative images of SA‐β‐gal staining in RAW264.7 macrophages from SiO_2_ and siAtf3 treatment, scale bar = 100 µm. Each experiment was performed in triplicate to ensure reproducibility of results. (L,M) Western blot and quantitative analysis of fibrotic markers (Col I and α‐SMA) in NIH/3T3 cells, all data were expressed as the means ± SD of at least 3 independent experiments, with ^****^ *p* < 0.0001 vs. the indicated group, and *p* values were from a 1‐way ANOVA post–hoc test with Tukey's correction. (N) Fibroblast contraction was measured using the collagen gel‐based contraction assay. Each experiment was performed in triplicate to ensure reproducibility of results. (O) Representative micrographs of cell migration in the transwell migration assay. Each experiment was performed in triplicate to ensure reproducibility of results. (P) Interactions numbers and strength between the different subpopulations from scRNA‐seq data of normal and silicotic human lung tissues (n = 3 for each group). (Q) Strategy for the administration of AAV9‐Atf3 in the silica‐induced pulmonary fibrosis mouse model. (R, S) Western blot and quantitative analysis of ATF3 in mouse lung tissues from the AAV9‐ negative control (NC) and AAV9‐Atf3 treated group after silica administration, all data were expressed as the means ± SD of at least 3 independent experiments, with ^*^ *p* < 0.05 vs. the indicated group, and *p* values were from a 2‐tailed unpaired Student's *t*‐test. (T, U) SA‐β‐gal staining and positive area rate in mouse lung tissues from the AAV9‐NC and AAV9‐Atf3 treated group after silica administration, all data were expressed as the means ± SD of at least 3 independent experiments, with ^**^ *p* < 0.01 vs. the indicated group, and *p* values were from a 2‐tailed unpaired Student's *t*‐test. (V, W) Western blot and quantitative analysis of cellular senescence markers (P16 and P21) in mouse lung tissues from the AAV9‐NC and AAV9‐Atf3 treated group after silica administration, all data were expressed as the means ± SD of at least 3 independent experiments, with ^*^ *p* < 0.05 vs. the indicated group, and *p* values were from a 1‐way ANOVA post–hoc test with Tukey's correction. (X) Representative histopathological images of H&E, Masson, and Sirius red staining of mouse lung tissues from the AAV9‐NC and AAV9‐Atf3 treated group after silica administration (n = 3 for each group, biological replicates); scale bar = 20 µm. (Y, Z) Western blot and quantitative analysis of fibrotic markers (Col I and α‐SMA) in mouse lung tissues from the AAV9‐NC and AAV9‐Atf3 treated group after silica administration, all data were expressed as the means ± SD of at least 3 independent experiments, with ^*^ *p* < 0.05 and ^**^ *p* < 0.01 vs. the indicated group, and p values were from a 1‐way ANOVA post–hoc test with Tukey's correction.

To determine the key molecule involved in SiO_2_‐induced macrophage senescence, macrophages were further re‐clustered into 14 subsets based on our previous scRNA‐seq from PBS and silica‐treated mouse lungs (Figure S2A). Clusters 0, 2 and 11 exhibited higher expression of Cdkn1a, and surprisingly, compared to the control group, the expression of these genes (Ahr, Nfkbia, Runx1, Cebpa, Fabp1, Atf3, Jun and Egr1) was upregulated in the silica group (Figure 2A). Furthermore, our scRNA‐seq and bulk RNA‐seq data indicated a significant positive correlation between Runx1, Cebpa, and Atf3 expression and the expression of Cdkn1a in macrophages and lung tissues, respectively (Figure 2B,C). Notably, quantitative real‐time polymerase chain reaction (qRT‐PCR) and western blot analysis showed that only Atf3, but not Runx1 or Cebpa, was significantly upregulated in mouse fibrotic lung tissues (Figure S2B–E). Besides, qRT‐PCR analysis confirmed that Atf3 was, indeed, prominently highly expressed in SiO_2_‐stimulated macrophages, rather than other fibrotic lung effector cells (Figure S2F–I). These findings were corroborated by immunofluorescence staining of ATF3 and P21 from control and silica‐treated mouse lungs, and were evident from SiO_2_‐treated macrophages (Figure S2J–M). Publicly available scRNA‐seq data of human normal and silicotic lung tissues also exhibited the higher expression of ATF3 in silicotic lung macrophages (Figure 2D,E). Accordingly, we observed the overexpression of ATF3 and its co‐localization with P21 in human silicotic lung tissues (Figure 2F–H). In line with the above results, higher ATF3 expression in macrophages of IPF samples was also observed in publicly available scRNA‐seq data of IPF (GSE128033) (Figure S2N,O).

We subsequently evaluated the intervention effects of Atf3 in the SiO_2_‐induced macrophage senescence and murine model of silicosis. Treatment with Atf3 siRNA in SiO_2_‐exposed macrophages significantly alleviated cellular senescence phenotypes (Figure 2I–K). Furthermore, we collected the supernatant from Atf3‐knockdown macrophages to treat fibroblasts (NIH/3T3 cells). Conditioned medium from SiO_2_ induced fibroblast activation, contraction, and migration, whereas Atf3 siRNA treatment suppressed these changes (Figure 2L–O). Moreover, publicly available scRNA‐seq data (both from silicosis and IPF) confirmed that stronger interaction between ATF3^+^ macrophages and fibroblasts (Figure 2P and Figure S2P). In addition, we generated macrophages specific to Atf3 knockdown by intratracheal delivery of adeno‐associated virus 9 (AAV9)‐Atf3, followed by silica exposure (Figure 2Q). Western blot assay showed a clear reduction of ATF3 protein in whole‐lung lysates after intervention with AAV9‐Atf3 (Figure 2R,S). Besides, Atf3 knockdown downregulated SA‐β‐gal activity and the expressions of the senescence‐associated markers (P16 and P21) in lung tissues (Figure 2T–W and Figure S2Q). Meanwhile, treatment with AAV9‐Atf3 significantly reduced lung injury and collagen accumulation, accompanied by improved overall lung appearance, lung coefficient, and decreased fibrosis marker expression (Figure 2X–Z and Figure S2R–X). Collectively, these findings suggest that ATF3 is up‐regulated in senescent macrophages and is a potential intervention target in a SiO_2_‐induced murine model of PF.

### 2.3 SPP1‐CD44 Signaling Pathway Mediates the Crosstalk of Senescent Macrophages and Fibroblasts

**FIGURE 3:**
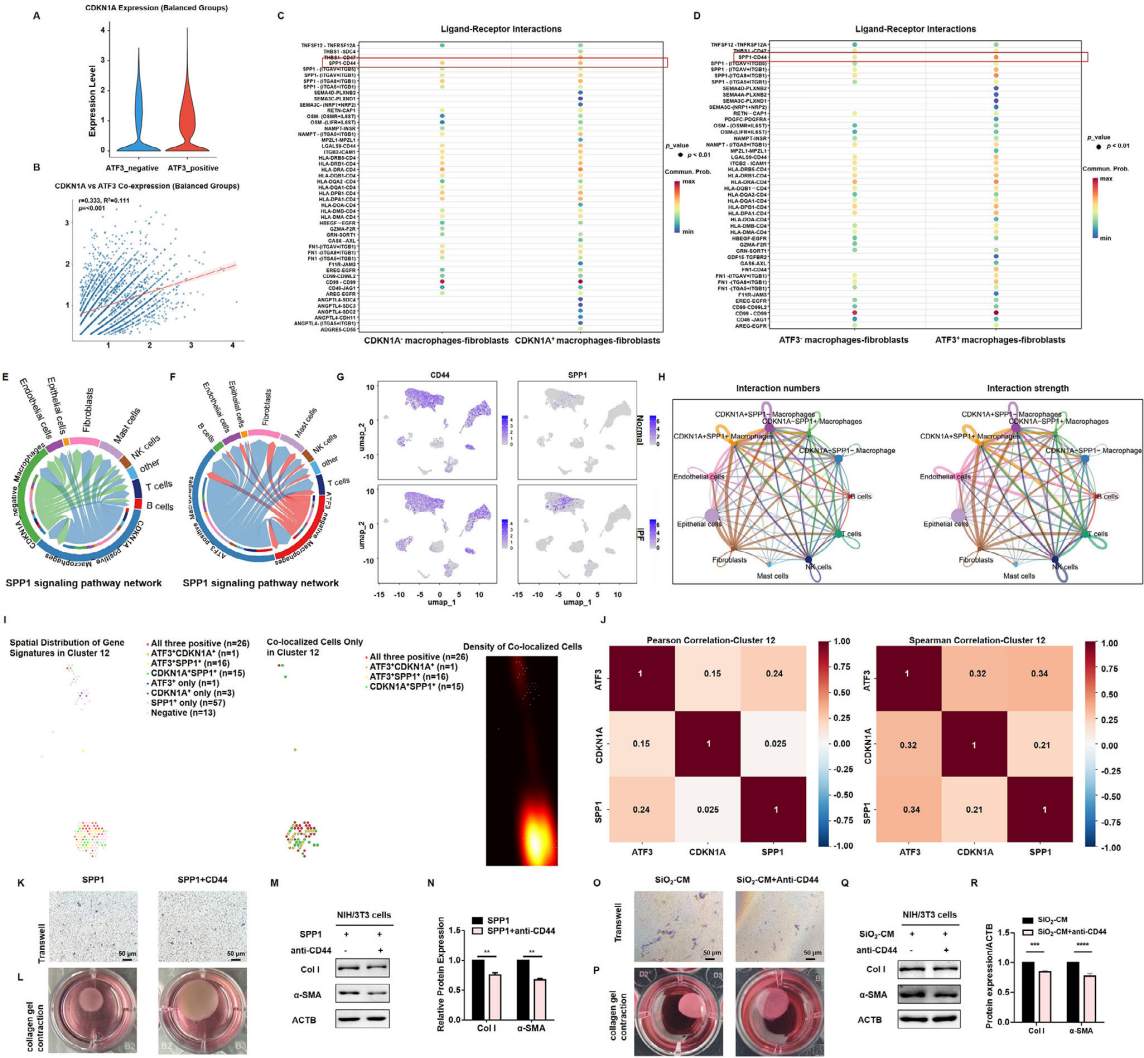
SPP1‐CD44 signaling pathway mediates the crosstalk of senescent macrophages and fibroblasts. (A) Mean CDKN1A expression levels in ATF3‑positive and ATF3‑negative cell groups from scRNA‐seq data (GSE128033) of normal and IPF lung tissues (n = 7 for the normal group and n = 8 for the IPF group, biological replicates). (B) Scatter plot of CDKN1A versus ATF3 co‑expression from scRNA‐seq data (GSE128033) of normal and IPF lung tissues (n = 7 for the normal group and n = 8 for the IPF group, biological replicates). (C) Overview of ligand‐receptor interactions between CDKN1A^−^ macrophages‐fibroblasts and CDKN1A^+^ macrophages‐fibroblasts from scRNA‐seq data (GSE128033) of normal and IPF lung tissues (n = 7 for the normal group and n = 8 for the IPF group, biological replicates). (D) Overview of ligand‐receptor interactions between ATF3^−^ macrophages‐fibroblasts and ATF3^+^ macrophages‐fibroblasts from scRNA‐seq data (GSE128033) of normal and IPF lung tissues (n = 7 for the normal group and n = 8 for the IPF group, biological replicates). (E) SPP1 signaling pathway network between CDKN1A^+^ macrophages and fibroblasts from scRNA‐seq data (GSE128033) of normal and IPF lung tissues (n = 7 for the normal group and n = 8 for the IPF group, biological replicates). (F) SPP1 signaling pathway network between ATF3^+^ macrophages and fibroblasts from scRNA‐seq data (GSE128033) of normal and IPF lung tissues (n = 7 for the normal group and n = 8 for the IPF group, biological replicates). (G) UMAP plots show CD44 and SPP1 expression in normal and IPF human lungs from scRNA‐seq data (GSE128033) of normal and IPF lung tissues (n = 7 for the normal group and n = 8 for the IPF group, biological replicates). (H) Interaction numbers and strength between the different subpopulations from scRNA‐seq data (GSE128033) of normal and IPF lung tissues (n = 7 for the normal group and n = 8 for the IPF group, biological replicates). (I) Heatmap of ATF3/ SPP1/ CDKN1A spot‐level co‐expression intensity from scRNA‐seq data (GSE128033) of normal and IPF lung tissues (n = 7 for the normal group and n = 8 for the IPF group, biological replicates). (J) Pearson and Spearman correlation matrices for the three genes from scRNA‐seq data (GSE128033) of normal and IPF lung tissues (n = 7 for the normal group and n = 8 for the IPF group, biological replicates). (K) Representative micrographs of cell migration in the transwell migration assay after SPP1 and anti‐CD44 antibody treatment. Each experiment was performed in triplicate to ensure reproducibility of results. (L) Fibroblast contraction was measured using the collagen gel‐based contraction assay after SPP1 and anti‐CD44 antibody treatment. Each experiment was performed in triplicate to ensure reproducibility of results. (M,N) Western blot and quantitative analysis of fibrotic markers (Col I and α‐SMA) in NIH/3T3 cells after SPP1 and anti‐CD44 antibody treatment. All data were expressed as the means ± SD of at least 3 independent experiments, with ^**^ *p* < 0.01 vs. the indicated group, and p values were from a 1‐way ANOVA post–hoc test with Tukey's correction. (O) Representative micrographs of cell migration in the transwell migration assay after SiO_2_‐CM and anti‐CD44 antibody treatment. Each experiment was performed in triplicate to ensure reproducibility of results. (P) Fibroblast contraction was measured using the collagen gel‐based contraction assay after SiO_2_‐CM and anti‐CD44 antibody treatment. Each experiment was performed in triplicate to ensure reproducibility of results. (Q‐R) Western blot and quantitative analysis of fibrotic markers (Col I and α‐SMA) in NIH/3T3 cells after SiO_2_‐CM and anti‐CD44 antibody treatment. All data were expressed as the means ± SD of at least 3 independent experiments, with ^***^ *p* < 0.001 and ^****^ *p* < 0.0001 vs. the indicated group, and *p* values were from a 1‐way ANOVA post–hoc test with Tukey's correction.

Next, we tried to explore how senescent macrophages promoted fibroblast activation and subsequently mediated the progression of PF. We analyzed publicly available scRNA‐seq data (GSE128033) from normal and IPF lung tissues, and found that a significant positive correlation exists between the expression of the ATF3 and CDKN1A in macrophages (Figure 3A,B). Surprisingly, intercellular communication analysis from scRNA‐seq data (GSE128033) demonstrated that the robust ligand‐receptor pairs between CDKN1A^+^ macrophages‐fibroblasts and ATF3^+^ macrophages‐fibroblasts, mainly through SPP1‐CD44 interaction axis (Figure 3C,D). Consistently, the SPP1 signaling pathway mediated more prominent crosstalk between CDKN1A^+^ macrophages and fibroblasts as well as between ATF3^+^ macrophages and fibroblasts (Figure 3E,F). Consistent with the transcriptomics results, the scRNA‐seq dataset (GSE128033) also showed the crosstalk of the SPP1‐CD44 signaling pathway between macrophages and fibroblasts (Figure 3G) and stronger interaction between CDKN1A^+^ SPP1^+^ Macrophages and fibroblasts (Figure 3H).

To systematically identify genes with significant spatial patterns in IPF, we first performed Moran's I spatial autocorrelation analysis on IPF spatial transcriptomics data. This metric quantifies whether gene expression is randomly distributed or exhibits clustered/dispersed patterns across tissue coordinates. Genes with high positive Moran's I values demonstrated significant spatial aggregation, suggesting their localization within specific histological microenvironments. The top 20 genes ranked by Moran's I value are listed in Table S5. Of note, SPP1 was highly enriched in Cluster 12 (Figure S3B,E). Based on this initial observation, we further employed multiple spatial analysis methods to comprehensively characterize the fibrotic microenvironment. Neighborhood enrichment analysis revealed preferential spatial co‐localization between distinct cell populations within the fibrotic niche, with higher enrichment scores indicating stronger co‐localization tendencies; for instance, macrophages and fibroblasts exhibited high enrichment scores (Figure S3A,D). To assess the spatial correlation of macrophages across different scales, we performed multi‐scale co‐occurrence analysis and found that within a distance of 200–400 µm, fibroblasts (Cluster 8) were more likely to appear in proximity to macrophages (value > 1), indicating significant co‐localization at this scale (Figure S3C). Integrating our previous single‐cell sequencing data, we observed enhanced SPP1‐CD44 interaction in both senescent and ATF3‐positive macrophage subpopulations. To validate this finding at the tissue level, we conducted spatial proximity analysis of SPP1 and CD44 expression in the spatial transcriptomics dataset, revealing a spatial basis for potential ligand‐receptor interaction (Figure S3E,F). Furthermore, we performed co‐localization analysis of ATF3, CDKN1A, and SPP1, which showed that regions positive for all three markers accounted for 19.2% of the analyzed area (Figure 3I), with significant positive correlations among their expression levels (Figure 3J). The migration, contraction, and activation of fibroblasts were increased when cultured with the conditioned medium of the SPP1‐treated macrophages, and anti‐CD44 intervention reduced these phenotypes (Figure S3G–J). Next, a more critical question is whether SPP1 can directly activate fibroblasts via CD44, rather than relying solely on an indirect co‐culture approach using conditioned medium from SPP1‐treated RAW264.7 macrophages. Notely, treatment with SPP1 plus a CD44‐neutralizing antibody in NIH/3T3 cells significantly attenuated fibroblast migration, contraction, and activation compared to treatment with SPP1 alone (Figure 3K–N). Besides, anti‐CD44 treatment also reversed the SiO_2_‐induced above phenotypes (Figure 3O–R). These data suggest that the SPP1‐CD44 signaling pathway involves the crosstalk of senescent macrophages and fibroblasts, thereby promoting fibroblast activation.

### 2.4 Atf3 Transcriptional Regulation of Mgarp Mediates Mitochondrial Damage, Macrophage Senescence, and Pulmonary Fibrosis

**FIGURE 4:**
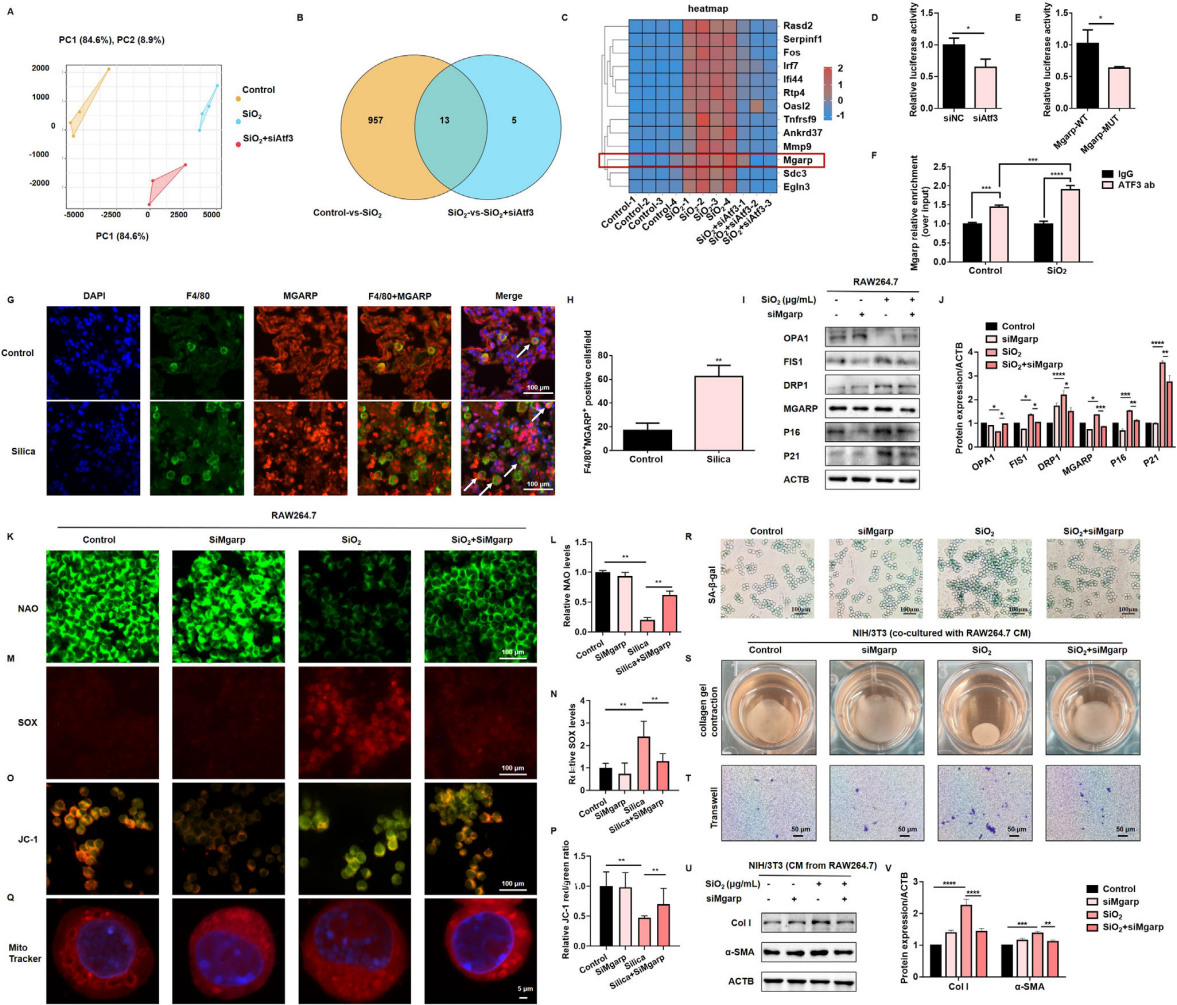
Atf3 transcriptional regulation of Mgarp mediates mitochondrial damage, macrophage senescence, and pulmonary fibrosis. (A) PCA shows the merged distribution of samples from control, SiO_2_ and SiO_2_ plus siAtf3‐treated macrophages. (B) Venn diagram of genes differentially expressed in the indicated groups. (C) Heatmap of differentially expressed genes (DEGs) from transcriptome sequencing analysis. (D) The dual‐luciferase reporter gene assay was performed to detect the relative luciferase activity of the wild‐type Mgarp promoter in RAW264.7 macrophages after siNC or siAtf3 treatment. All data were expressed as the means ± SD of at least 3 independent experiments, with ^*^ *p* < 0.05 vs the indicated group, and p values were from a 2‐tailed unpaired Student's *t*‐test. (E) The dual‐luciferase reporter gene assay was performed to detect the relative luciferase activity of the Mgarp promoter in RAW264.7 macrophages after Mgarp‐WT or Mgarp‐MUT treatment. All data were expressed as the means ± SD of at least 3 independent experiments, with ^*^ *p* < 0.05 vs the indicated group, and *p* values were from a 2‐tailed unpaired Student's *t*‐test. (F) Chromatin was harvested for immunoprecipitation with IgG, an anti‐ATF3 antibody in the indicated groups. The expression of Mgarp relative enrichment was detected by qRT‐PCR analysis. All data were expressed as the means ± SD of at least 3 independent experiments, with ^***^ *p* < 0.001 and ^****^ *p* < 0.0001 vs. the indicated group, and *p* values were from a 1‐way ANOVA post–hoc test with Tukey's correction. (G) Representative results for coimmunostaining and quantitative analysis of F4/80 and MGARP in control and silica‐induced mouse lung sections. Green represents F4/80, red represents MGARP, and blue represents nuclear DNA staining by DAPI. Scale bar = 100 µm. Each experiment was performed in triplicate to ensure reproducibility of results. (H) F4/80 and MGARP‐positive cell from the lung sections of control and silica groups, all data were expressed as the means ± SD of at least 3 independent experiments, with ^**^ *p* < 0.01 vs. the indicated group and p values were from a 2‐tailed unpaired Student's *t*‐test. (I,J) Western blot and quantitative analysis of mitochondrial damage markers (DRP1, FIS1, OPA1) and cellular senescence markers (P16 and P21) in RAW264.7 macrophages after SiO_2_ and siMgarp treatment. All data were expressed as the means ± SD of at least 3 independent experiments, with ^*^ *p* < 0.05, ^**^ *p* < 0.01, ^***^ *p* < 0.001, and ^****^ *p* < 0.0001 vs. the indicated group, and *p* values were from a 1‐way ANOVA post hoc test with Tukey's correction. (K, L) Representative images of NAO staining and relative NAO fluorescence quantification in RAW264.7 macrophages after SiO_2_ and siMgarp treatment. Scale bar = 100 µm. Each experiment was performed in triplicate to ensure reproducibility of results. All data were expressed as the means ± SD of at least 3 independent experiments, with ^**^ *p* < 0.01 vs. the indicated group, and *p* values were from a 1‐way ANOVA post–hoc test with Tukey's correction. (M, N) Representative images of SOX staining and relative SOX fluorescence quantification in RAW264.7 macrophages after SiO_2_ and siMgarp treatment. Scale bar = 100 µm. Each experiment was performed in triplicate to ensure reproducibility of results. All data were expressed as the means ± SD of at least 3 independent experiments, with ^**^ *p* < 0.01 vs. the indicated group, and *p* values were from a 1‐way ANOVA post hoc test with Tukey's correction. (O, P) Representative images of JC‐1 staining and relative JC‐1 fluorescence quantification in RAW264.7 macrophages after SiO_2_ and siMgarp treatment. Scale bar = 100 µm. Each experiment was performed in triplicate to ensure reproducibility of results. All data were expressed as the means ± SD of at least 3 independent experiments, with ^**^ *p* < 0.01 vs. the indicated group, and *p* values were from a 1‐way ANOVA post–hoc test with Tukey's correction. (Q) Representative images of MitoTracker staining in RAW264.7 macrophages after SiO_2_ and siMgarp treatment. Scale bar = 5 µm. Each experiment was performed in triplicate to ensure reproducibility of results. (R) Representative images of SA‐β‐gal staining in RAW264.7 macrophages after SiO_2_ and siMgarp treatment. Scale bar = 100 µm. Each experiment was performed in triplicate to ensure reproducibility of results. (S) Fibroblast contraction was measured using the collagen gel‐based contraction assay. Each experiment was performed in triplicate to ensure reproducibility of results. (T) Representative micrographs of cell migration in the transwell migration assay. Each experiment was performed in triplicate to ensure reproducibility of results. (U, V) Western blot and quantitative analysis of fibrotic markers (Col I and α‐SMA) in NIH/3T3 cells, all data were expressed as the means ± SD of at least 3 independent experiments, with ^***^ *p* < 0.001 and ^****^ *p* < 0.0001 vs. the indicated group, and p values were from a 1‐way ANOVA post–hoc test with Tukey's correction.

Since ATF3 is an important transcription factor, we next sought to identify the downstream transcriptional target of ATF3 in SiO_2_‐stimulated senescent macrophages. Specifically, we performed RNA transcriptome sequencing from control, SiO_2_ and SiO_2_ plus siAtf3 treated macrophages, and the principal component analysis (PCA) showed good separation between the samples from three groups (Figure 4A). There were 13 overlapping genes among three groups in the venn diagram, and the differential expression patterns of these genes were further illustrated in a corresponding heatmap (Figure 4B,C). Among these 13 genes, we selected Mgarp as a downstream transcriptional target of Atf3. This choice was driven by both the positive correlation of ATF3 with the mitochondrial fission‐related proteins mitochondrial fission 1 protein (FIS1) and dynamin‐related protein 1 (DRP1) in scRNA‐seq data from human and mouse silicotic lung tissues (Figure S4A–D) and the established close link between mitochondrial function and cellular senescence. To clarify the direct regulation of Mgarp transcription by Atf3, we performed luciferase reporter assays. RAW264.7 macrophages were transfected with the wild‐type Mgarp promoter reporter, together with negative control siRNA (siNC) or siAtf3. Knockdown of Atf3 significantly decreased the activity of the wild‐type Mgarp promoter (Figure 4D). Next, RAW264.7 macrophages were transfected with the wild‐type Mgarp promoter reporter (Mgarp‐WT) or a reporter containing a mutation in the predicted ATF3 binding site (Mgarp‐MUT). Consistently, the luciferase activity of the Mgarp‐MUT reporter was markedly lower than that of Mgarp‐WT (Figure 4E). Subsequent ChIP‐PCR, molecular docking using AlphaFold3, and qRT‐PCR assays further confirmed the regulatory role of Atf3 on Mgarp (Figure 4F and Figure S4E–H).

We then determined the role of Mgarp in regulating mitochondrial function and macrophage senescence. Mgarp expression was increased, and immunofluorescence staining showed that its expression was co‐localized with F4/80 in silicotic mouse lung tissues (Figure 4G,H and Figure S4I,J). SiO_2_ induced the abnormal expression of mitochondrial injury and senescence‐related proteins of macrophages, which was significantly reversed by Mgarp siRNA intervention (Figure 4I,J and Figure S4K–R). Similar to the mitochondrial injury‐related protein expression results, staining with 10‐Nonyl Acridine orange (NAO), MitoSOX Red (SOX), JC‐1, and MitoTracker indicated that SiO_2_‐induced mitochondrial damage could be rescued by Mgarp siRNA and Atf3 siRNA (Figure 4K–Q and Figure S4S–Y). Importantly, Atf3 siRNA also restored the abnormal expression of mitochondrial fission and fusion‐related proteins by rescuing MGARP expression (Figure S4Z–AA). In addition, Mgarp siRNA treatment also reversed SiO_2_‐induced increased SA‐β‐gal activity of RAW264.7 macrophages (Figure 4R). Finally, we collected the supernatant from Mgarp‐knockdown RAW264.7 macrophages to stimulate fibroblasts, and found that SiO_2_ significantly increased fibroblast contraction, migration, and activation, whereas Mgarp siRNA treatment suppressed these phenotypes (Figure 4S–V).

### 2.5 SIRT6‐Mediated ATF3 Deacetylation Facilitates its Nuclear Transport in Senescent Macrophages

**FIGURE 5:**
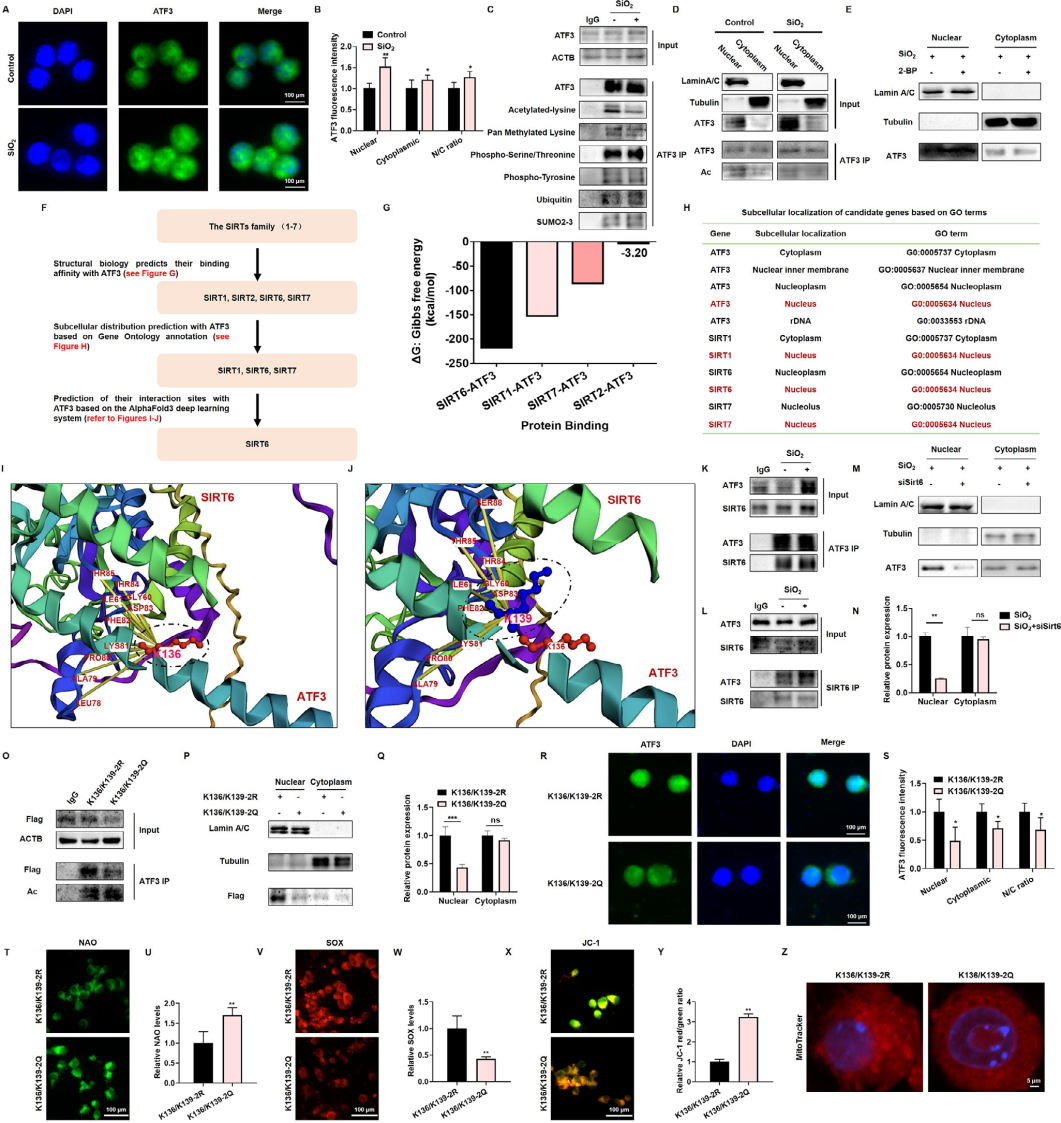
SIRT6‐mediated ATF3 deacetylation facilitates its nuclear transport in senescent macrophages. (A) Immunofluorescence staining of ATF3 in RAW264.7 macrophages from the control and SiO_2_‐treated groups. Green represents ATF3, blue represents nuclear DNA staining by DAPI, scale bar = 100 µm. Each experiment was performed in triplicate to ensure reproducibility of results. (B) ATF3 fluorescence intensity was measured after control and SiO_2_ treatment. At least 30 cells per group from 3 random fields were analyzed in three independent experiments, and all data were expressed as the means ± SD, with ^*^ *p* < 0.05 and ^**^ *p* < 0.01 vs. the indicated group, and *p* values were from a 1‐way ANOVA post–hoc test with Tukey's correction. (C) RAW264.7 macrophages were treated with or without SiO_2_, then were immunoprecipitated with anti‐ATF3 antibody, followed by western blot assay of immunocomplexes with the indicated antibody. Each experiment was performed in triplicate to ensure reproducibility of results. (D) RAW264.7 macrophages were exposed to SiO_2_ or not. Acetylation of ATF3 and ATF3 in the nucleus or cytoplasm was detected by immunoprecipitation with an anti‐acetylation antibody. Each experiment was performed in triplicate to ensure reproducibility of results. (E) The ATF3 expression in the nucleus or cytoplasm was detected by western blot in the indicated groups. Each experiment was performed in triplicate to ensure reproducibility of results. (F) Flowchart of stepwise screening strategy used to identify SIRT6 as the target of interest from the SIRT family members SIRT1 through SIRT7. (G) The histogram displays the Gibbs free energy results between the indicated groups. Each data point was performed in triplicate to ensure reproducibility of results. (H) Subcellular localization of candidate genes (including ATF3, SIRT1, SIRT6, and SIRT7) based on GO terms. (I, J) Potential binding sites between SIRT6 and ATF3 (including K136 and K139 in ATF3) were predicted based on molecular docking. (K, L) RAW264.7 macrophages were treated with or without SiO_2_, then were immunoprecipitated with anti‐ATF3 or SIRT6 antibody, followed by western blot assay of immunocomplexes with the indicated antibody. Each experiment was performed in triplicate to ensure reproducibility of results. (M, N) The ATF3 expressions and quantitative analysis in the nucleus or cytoplasm were detected by western blot in the indicated groups; all data were expressed as the means ± SD of at least 3 independent experiments, with ^**^ *p* < 0.01 vs. the indicated group, and *p* values were from a 1‐way ANOVA post–hoc test with Tukey's correction. (O) RAW264.7 macrophages were treated with K136/K139‐2R or K136/K139‐2Q, then were immunoprecipitated with Flag antibody, followed by western blot assay of immunocomplexes with the indicated antibody. Each experiment was performed in triplicate to ensure reproducibility of results. (P, Q) The ATF3 expressions and quantitative analysis in the nucleus or cytoplasm were detected by western blot in the indicated groups; all data were expressed as the means ± SD of at least 3 independent experiments, with ^***^ *p* < 0.001 vs. the indicated group, and *p* values were from a 1‐way ANOVA post–hoc test with Tukey's correction. (R) Immunofluorescence staining of ATF3 in RAW264.7 macrophages for the indicated groups. Green represents ATF3, blue represents nuclear DNA staining by DAPI, scale bar = 100 µm. Each experiment was performed in triplicate to ensure reproducibility of results. (S) ATF3 fluorescence intensity was measured after K136/K139‐2R and K136/K139‐2Q treatment. At least 30 cells per group from 3 random fields were analyzed in three independent experiments, and all data were expressed as the means ± SD, with ^*^ *p* < 0.05 vs. the indicated group, and *p* values were from a 1‐way ANOVA post–hoc test with Tukey's correction. (T, U) Representative images of NAO staining and relative NAO fluorescence quantification in RAW264.7 macrophages after K136/K139‐2R and K136/K139‐2Q treatment, scale bar = 100 µm. Each experiment was performed in triplicate to ensure reproducibility of results. All data were expressed as the means ± SD of at least 3 independent experiments, with ^**^ *p* < 0.01 vs. the indicated group, and p values were from a 2‐tailed unpaired Student's *t*‐test. (V, W) Representative images of SOX staining and relative SOX fluorescence quantification in RAW264.7 macrophages after K136/K139‐2R and K136/K139‐2Q treatment, scale bar = 100 µm. Each experiment was performed in triplicate to ensure reproducibility of results. All data were expressed as the means ± SD of at least 3 independent experiments, with ^**^ *p* < 0.01 vs. the indicated group, and p values were from a 2‐tailed unpaired Student's *t*‐test. (X‐Y) Representative images of JC‐1 staining and relative JC‐1 fluorescence quantification in RAW264.7 macrophages after K136/K139‐2R and K136/K139‐2Q treatment, scale bar = 100 µm. Each experiment was performed in triplicate to ensure reproducibility of results. All data were expressed as the means ± SD of at least 3 independent experiments, with ^**^ *p* < 0.01 vs. the indicated group, and *p* values were from a 2‐tailed unpaired Student's *t*‐test. (Z) Representative images of MitoTracker staining in RAW264.7 macrophages after K136/K139‐2R and K136/K139‐2Q treatment, scale bar = 5 µm. Each experiment was performed in triplicate to ensure reproducibility of results.

In addition to transcriptional regulation, it should be noted that the complex mechanisms of ATF3 regulating macrophage senescence merit in‐depth exploration. Based on the immunofluorescence staining, we found that SiO_2_ promoted ATF3 to enter the nucleus (Figure 5A,B). Hence, a central focus of further investigation is to elucidate the mechanism through which enhanced ATF3 nuclear translocation activates Mgarp transcription, leading to the process of cellular senescence. Previous studies have shown that multiple post‐translational modifications, such as phosphorylation, acetylation, methylation, ubiquitination, SUMOylation, and palmitoylation, can affect the nuclear translocation of proteins [22, 23, 24, 25, 26]. Control and SiO_2_‐treated RAW264.7 macrophages were collected and immunoprecipitated with anti‐ATF3. Then, the immunoprecipitates were analyzed by western blotting with antibodies against the above specific post‐translational modifications. The results of immunoprecipitation showed that ATF3 acetylation was significantly lower and had differential distribution between the cytoplasm and the nucleus in SiO_2_‐induced macrophages, while the other modifications, including phosphorylation, methylation, ubiquitination, and SUMOylation, showed no significant changes (Figure 5C,D). Furthermore, the palmitoylation inhibitor 2‐bromopalmitic acid (2‐BP) did not affect the nuclear‐cytoplasmic distribution of ATF3, thus ruling out the regulation of ATF3 nuclear translocation by palmitoylation (Figure 5E).

Since ATF3 acetylation was reduced upon SiO_2_ treatment, we sought to identify the specific deacetylase regulating ATF3 deacetylation. It is well known that deacetylation is mediated by sirtuins (SIRTs) and histone deacetylases (HDACs) [27]. We used trichostatin A (TSA, inhibitor of histone deacetylase HDAC classes I, II, and IV) and nicotinamide (NAM, inhibitor of the SIRT family deacetylases) to treat SiO_2_‐exposed RAW264.7 macrophages and found that both agents could restore ATF3 acetylation (Figure S5A,B). Existing evidence indicates that HDACs regulate ATF3 acetylation [28]; however, whether the sirtuin family deacetylases modulate this modification has not yet been reported. Therefore, we performed a computational analysis pipeline (Figure 5F). First, analysis of the Gibbs free energy of binding between SIRT family members and ATF3 preliminarily identified SIRT1, SIRT2, SIRT6, and SIRT7 as potential interactors (Figure 5G). Subsequent Gene Ontology (GO) annotation of subcellular localization revealed nuclear co‑localization of ATF3 with SIRT1, SIRT6, and SIRT7 (Figure 5H). Finally, molecular docking using AlphaFold3, a deep learning‑based protein structure prediction system [29], demonstrated that only SIRT6 specifically recognizes and binds to ATF3 at lysine residues K136 and K139 (Figure 5I,J). Subsequent co‐immunoprecipitation (Co‐IP) and western blot experiments confirmed the binding of SIRT6 and ATF3, and SIRT family broad‐spectrum inhibitor NAM affected their expression (Figure 5K,L and Figure S5C,D). More importantly, SiO_2_‐induced nuclear translocation of ATF3 was reduced in Sirt6 knockdown RAW264.7 macrophages, which was confirmed by western blot analyses and immunofluorescence assays (Figure 5M,N and Figure S5E,F).

Next, we further investigated whether alterations in the acetylation level of ATF3 affect its nuclear translocation and subsequent functions. We then constructed lysine mutants of ATF3, including K136/K139‐2R, a deacetylation mimic mutant; and K136/K139‐2Q, an acetylation mimic mutant (Figure 5O and Figure S5G). Notably, RAW264.7 macrophages treated with the ATF3 deacetylation mimic mutant exhibited significantly increased ATF3 nuclear translocation (Figure 5P–S). Additionally, the ATF3 deacetylation mimic mutant significantly alleviated SiO_2_‐induced mitochondrial damage and cellular senescence in RAW264.7 macrophages (Figure 5T–Z and Figure S5H,I). These results show that ATF3 deacetylation facilitates its nuclear translocation and subsequent functions in SiO_2_‐induced macrophage senescence.

### 2.6 Nuclear Transport Protein Importin α Mediates the Nuclear Transport of ATF3

**FIGURE 6:**
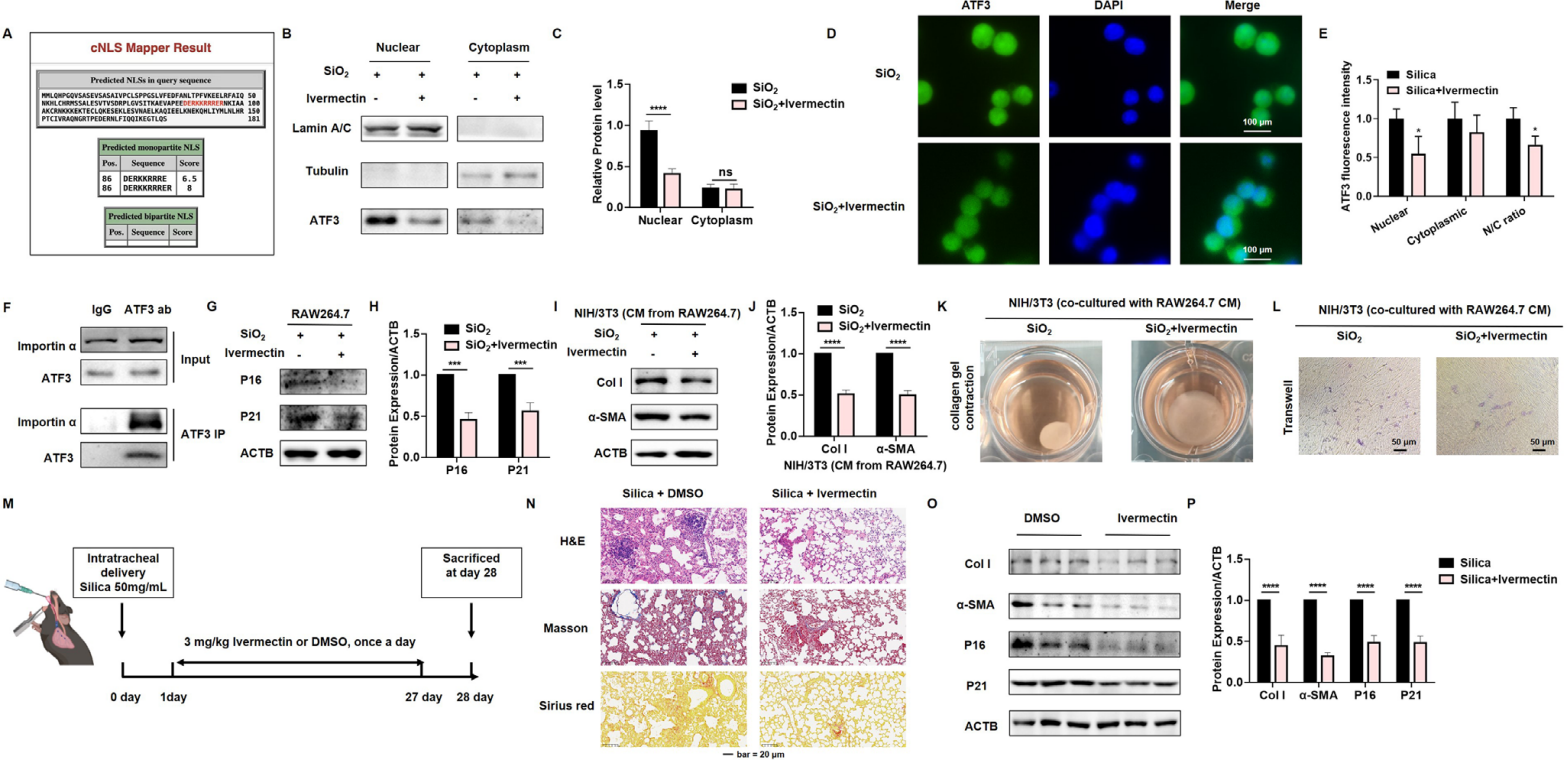
Nuclear transport protein importin α mediates the nuclear transport of ATF3. (A) cNLS Mapper (http://nls‐mapper.iab.keio.ac.jp/) identified the putative NLS sequences of ATF3. (B,C) The ATF3 expression and quantitative analysis in the nucleus or cytoplasm were detected by western blot in SiO_2_ or Ivermectin‐treated RAW264.7 macrophages. Each experiment was performed in triplicate to ensure reproducibility of results. All data were expressed as the means ± SD, with ^****^ *p* < 0.0001 vs. the indicated group, and p values were from a 1‐way ANOVA post–hoc test with Tukey's correction. (D) Immunofluorescence staining of ATF3 in SiO_2_ or Ivermectin‐treated RAW264.7 macrophages. Green represents ATF3, blue represents nuclear DNA staining by DAPI, scale bar = 100 µm. Each experiment was performed in triplicate to ensure reproducibility of results. (E) ATF3 fluorescence intensity was measured after SiO_2_ or Ivermectin treatment. At least 30 cells per group from 3 random fields were analyzed in three independent experiments, and all data were expressed as the means ± SD, with ^*^ *p* < 0.05 vs. the indicated group, and *p* values were from a 1‐way ANOVA post–hoc test with Tukey's correction. (F) Immunoprecipitation analysis with anti‐ATF3 antibody followed by western blot assay of immunocomplexes with the indicated antibody. Each experiment was performed in triplicate to ensure reproducibility of results. (G‐H) Western blot and quantitative analysis of cellular senescence markers (P16 and P21) in RAW264.7 macrophages after Ivermectin treatment, all data were expressed as the means ± SD of at least 3 independent experiments, with ^***^ *p* < 0.001 and ^****^ *p* < 0.0001 vs. the indicated group, and p values were from a 1‐way ANOVA post–hoc test with Tukey's correction. (I,J) Western blot and quantitative analysis of fibrotic markers (Col I and α‐SMA) in fibroblasts after Ivermectin treatment, all data were expressed as the means ± SD of at least 3 independent experiments, with ^****^ *p* < 0.0001 vs. the indicated group, and p values were from a 1‐way ANOVA post–hoc test with Tukey's correction. (K) Fibroblast contraction was measured using the collagen gel‐based contraction assay after Ivermectin treatment. Each experiment was performed in triplicate to ensure reproducibility of results. (L) Representative micrographs of cell migration in the transwell migration assay after Ivermectin treatment. Each experiment was performed in triplicate to ensure reproducibility of results. (M) Strategy for the administration of Ivermectin in the silica‐induced pulmonary fibrosis mouse model. (N) Representative histopathological images of H&E, Masson, and Sirius red staining of mouse lung tissues after Ivermectin treatment (n = 6 for each group, biological replicates); scale bar = 20 µm. Each experiment was performed in triplicate to ensure reproducibility of results. (O, P) Western blot and quantitative analysis of fibrotic markers (Col I and α‐SMA) and cellular senescence markers (P16 and P21) in mouse lung tissues after Ivermectin treatment, all data were expressed as the means ± SD of at least 3 independent experiments, with ^****^ *p* < 0.0001 vs. the indicated group, and *p* values were from a 1‐way ANOVA post–hoc test with Tukey's correction.

Beyond post‐translational modifications, the nucleocytoplasmic shuttling of many proteins is usually controlled by nuclear localization sequence (NLS) and NLS receptors [30]. Next, to explore whether ATF3 itself harbors an NLS, we used cNLS Mapper (http://nls‐mapper.iab.keio.ac.jp/) and identified the putative NLS sequences of ATF3 (Figure 6A). The importin α/β1 heterodimer mediates nuclear import of classical NLS‐bearing proteins, with importin α serving as the adaptor that specifically recognizes and binds the NLS sequence [31]. Thus, we employed importin α inhibitor, Ivermectin, to intervene in SiO_2_‐induced ATF3 nuclear translocation and found that Ivermectin treatment significantly decreased ATF3 nuclear translocation (Figure 6B–E). Besides, immunoprecipitation analysis further demonstrated the binding of importin α and ATF3 (Figure 6F).

We next investigated the role of Ivermectin in macrophage senescence and PF both in vitro and in vivo. Western blot and SA‐β‐gal staining results showed that treatment with Ivermectin attenuated SiO_2_‐induced macrophage senescence (Figure 6G,H and Figure S6A). Supernatant from SiO_2_ and Ivermectin‐treated RAW264.7 macrophages decreased fibroblast activation, contraction, and migration (Figure 6I–L). Further, we developed intervention models for mice with silica‐induced PF using Ivermectin (Figure 6M). Staining of pathological sections revealed that Ivermectin alleviated silica exposure‐induced lung injury and PF (Figure 6N). Moreover, treatment with Ivermectin mitigated silica‐stimulated PF as shown by the results of the appearance of the mouse lung, organ coefficient of the lung, and hydroxyproline content (Figure S6B–F). The expression of fibrotic and cellular senescence‐associated markers was also downregulated in the silicotic mouse lung tissues following interventions with Ivermectin (Figure 6O–P and Figure S6G,H).

### 2.7 HSP70 Impairs the Nuclear Import of ATF3 through Competitive Binding with Importin α

**FIGURE 7:**
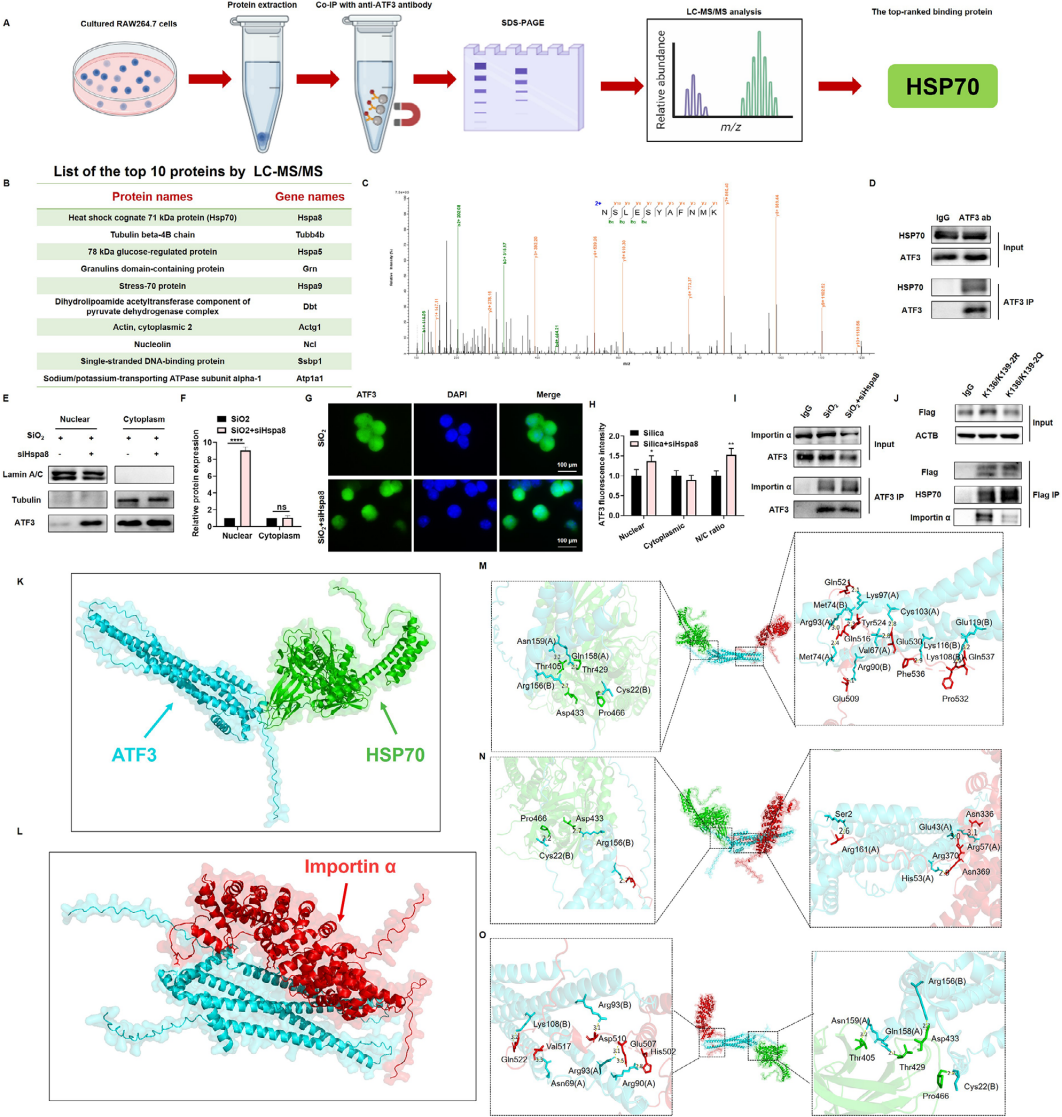
HSP70 impairs the nuclear import of ATF3 through competitive binding with Importin α. (A) Flowchart of identifying ATF3‐binding proteins (mainly HSP70) by LC‐MS/MS. (B) List of the top 10 ATF3‐binding proteins by LC‐MS/MS. (C) STRING analysis of the HSP70 protein obtained by mass spectrometry. (D) Immunoprecipitation analysis with anti‐ATF3 antibody followed by western blot assay of immunocomplexes with the indicated antibody. Each experiment was performed in triplicate to ensure reproducibility of results. (E, F) The ATF3 expressions and quantitative analysis in the nucleus or cytoplasm were detected by western blot in RAW264.7 macrophages treated with siHspa8; all data were expressed as the means ± SD of at least 3 independent experiments, with ^****^ *p* < 0.0001 vs. the indicated group, and *p* values were from a 1‐way ANOVA post– hoc test with Tukey's correction. (G) Immunofluorescence staining of ATF3 in RAW264.7 macrophages treated with siHspa8. Green represents ATF3, blue represents nuclear DNA staining by DAPI, scale bar = 100 µm. Each experiment was performed in triplicate to ensure reproducibility of results. (H) ATF3 fluorescence intensity was measured after SiO_2_ and siHspa8 treatment. At least 30 cells per group from 3 random fields were analyzed in three independent experiments, and all data were expressed as the means ± SD, with ^*^ *p* < 0.05 and ^**^ *p* < 0.01 vs. the indicated group, and *p* values were from a 1‐way ANOVA post hoc test with Tukey's correction. (I) Immunoprecipitation analysis with anti‐ATF3 antibody in RAW264.7 macrophages treated with siHspa8, followed by western blot assay of immunocomplexes with the indicated antibody. Each experiment was performed in triplicate to ensure reproducibility of results. (J) Immunoprecipitation analysis with Flag antibody in RAW264.7 macrophages treated with K136/K139‐2R or K136/K139‐2Q, followed by western blot assay of immunocomplexes with the indicated antibody. Each experiment was performed in triplicate to ensure reproducibility of results. (K, L) Schematic diagram of the docking results for ATF3, HSP70, and Importin α proteins. Displayed in Surface+Cartoon mode, the HSP70 protein is colored green, the ATF3 protein is colored cyan, and the Importin‐a protein is colored red. (M, O) Detailed diagram of local interactions in protein‐protein docking.

We next investigated whether there are other regulatory mechanisms for importin α‑mediated ATF3 nuclear translocation. We analyzed immunoprecipitates of RAW264.7 macrophage cell lysates incubated with an anti‑ATF3 antibody by high‑resolution LC‑MS/MS and found that HSP70 was the top‑ranked binding protein of ATF3 (Figure 7A,B). The representative MS/MS spectrum of a peptide matching validation of HSP70 is shown in Figure 7C. The antibody against ATF3 was able to pull down HSP70 in macrophages by immunoprecipitation (Figure 7D), confirming direct binding between ATF3 and HSP70.

To investigate the effect of HSP70 on ATF3 nuclear translocation, we knocked down HSP70 in RAW264.7 macrophages using siRNA (Figure S7A,B). Surprisingly, western blot analysis and immunofluorescence staining revealed that knocking down HSP70 promoted ATF3 nuclear translocation (Figure 7E–H). We therefore sought to determine how lower expression of HSP70 facilitates the nuclear translocation of ATF3. Remarkably, HSP70 was decreased in SiO_2_‐treated macrophages, and the Cycloheximide (CHX) assay demonstrated that downregulated HSP70 significantly decreased the rate of ATF3 protein degradation (Figure S7C–F). Previous studies have suggested that HSP70 serves as a molecular chaperone for the degradation of proteins [32]. However, knockdown of HSP70 significantly reduced the protein expression of the lysosomal‐associated membrane protein 1 (Lamp1) (Figure S7G–J). Consequently, we reasonably speculate that reduced HSP70 expression in senescent macrophages compromises lysosomal degradation, which in turn facilitates ATF3 nuclear translocation.

A key question is whether the facilitation of ATF3 nuclear translocation by HSP70 knockdown in macrophages is associated with importin α. Immunoprecipitation results showed that HSP70 knockdown significantly increased the interaction between ATF3 and importin α (Figure 7I). In addition, the acetylation status of ATF3 regulated the competition between HSP70 and importin α for binding to ATF3 (Figure 7J). This mechanism was further confirmed by molecular docking simulations (Figure 7K–O), which revealed that after the binding of ATF3 and importin α, the subsequent association of the complex with HSP70 resulted in a decrease in the highest binding confidence score from 0.88 to 0.80. Furthermore, a statistically significant difference was observed among the top five binding affinity scores (Figure S7K). Taken together, these findings suggest that HSP70 impairs the nuclear import of ATF3 by competing with importin α for binding to ATF3.

### 2.8 Itraconazole‐Mediated Inhibition of the ATF3‐Importin α Interaction Blocks ATF3 Nuclear Entry

**FIGURE 8:**
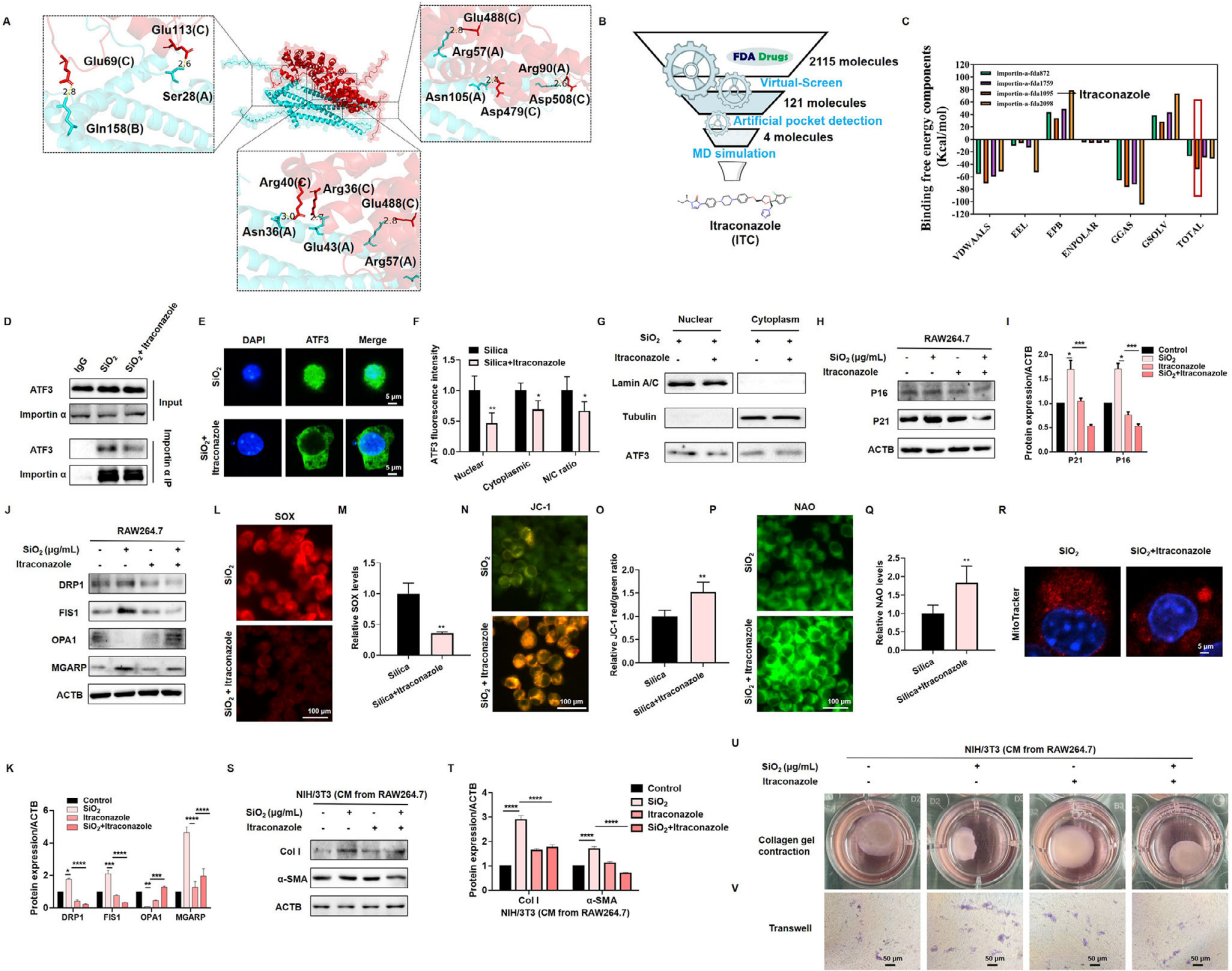
Itraconazole‐mediated inhibition of the ATF3‐Importin α interaction blocks ATF3 nuclear entry. (A) Detailed diagram of the interaction between ATF3 and Importin α proteins. (B) Virtual screening flowchart for pocket‐based integration. (C) The binding free energy of small molecule inhibitors on the interaction between ATF3 and Importin α. Each data point was performed in triplicate to ensure reproducibility of results. (D) Immunoprecipitation analysis with anti‐Importin α antibody in macrophages treated with Itraconazole, followed by western blot assay of immunocomplexes with the indicated antibody. Each experiment was performed in triplicate to ensure reproducibility of results. (E) Immunofluorescence staining of ATF3 in RAW264.7 macrophages treated with SiO_2_ and Itraconazole. Green represents ATF3, blue represents nuclear DNA staining by DAPI, scale bar = 5 µm. Each experiment was performed in triplicate to ensure reproducibility of results. (F) ATF3 fluorescence intensity was measured after SiO_2_ and Itraconazole treatment. At least 30 cells per group from 3 random fields were analyzed in three independent experiments, and all data were expressed as the means ± SD, with ^*^ *p* < 0.05 and ^**^ *p* < 0.01 vs. the indicated group, and *p* values were from a 1‐way ANOVA post–hoc test with Tukey's correction. (G) The ATF3 expression in the nucleus or cytoplasm was detected by western blot in the indicated groups. Each experiment was performed in triplicate to ensure reproducibility of results. (H, I) Western blot and quantitative analysis of cellular senescence markers (P16 and P21) in RAW264.7 macrophages after SiO_2_ and Itraconazole treatment. All data were expressed as the means ± SD of at least 3 independent experiments, with ^*^ *p* < 0.05 and ^***^ *p* < 0.001 vs. the indicated group, and *p* values were from a 1‐way ANOVA post–hoc test with Tukey's correction. (J, K) Western blot and quantitative analysis of mitochondrial damage markers (DRP1, FIS1, OPA1) and MGARP in RAW264.7 macrophages. All data were expressed as the means ± SD of at least 3 independent experiments, with ^*^ *p* < 0.05, ^**^ *p* < 0.01, ^***^ *p* < 0.001, and ^****^ *p* < 0.0001 vs. the indicated group, and *p* values were from a 1‐way ANOVA post–hoc test with Tukey's correction. (L, M) Representative images of NAO staining and relative NAO fluorescence quantification in RAW264.7 macrophages after SiO_2_ and Itraconazole treatment, scale bar = 100 µm. Each experiment was performed in triplicate to ensure reproducibility of results. All data were expressed as the means ± SD of at least 3 independent experiments, with ^**^ *p* < 0.01 vs. the indicated group, and *p* values were from a 2‐tailed unpaired Student's *t*‐test. (N,O) Representative images of SOX staining and relative SOX fluorescence quantification in RAW264.7 macrophages after SiO_2_ and Itraconazole treatment, scale bar = 100 µm. Each experiment was performed in triplicate to ensure reproducibility of results. All data were expressed as the means ± SD of at least 3 independent experiments, with ^**^ *p* < 0.01 vs. the indicated group, and p values were from a 2‐tailed unpaired Student's *t*‐test. (P, Q) Representative images of JC‐1 staining and relative JC‐1 fluorescence quantification in RAW264.7 macrophages after SiO_2_ and Itraconazole treatment, scale bar = 100 µm. Each experiment was performed in triplicate to ensure reproducibility of results. All data were expressed as the means ± SD of at least 3 independent experiments, with ^**^ *p* < 0.01 vs. the indicated group, and *p* values were from a 2‐tailed unpaired Student's *t*‐test. (R) Representative images of MitoTracker staining in RAW264.7 macrophages after SiO_2_ and Itraconazole treatment, scale bar = 5 µm. Each experiment was performed in triplicate to ensure reproducibility of results. (S, T) Western blot and quantitative analysis of fibrotic markers (Col I and α‐SMA) in NIH/3T3 cells, all data were expressed as the means ± SD of at least 3 independent experiments, with ^****^ *p* < 0.0001 vs. the indicated group, and *p* values were from a 1‐way ANOVA post–hoc test with Tukey's correction. (U) Fibroblast contraction was measured using the collagen gel‐based contraction assay after SiO_2_ and Itraconazole treatment. Each experiment was performed in triplicate to ensure reproducibility of results. (V) Representative micrographs of cell migration in the transwell migration assay after SiO_2_ and Itraconazole treatment. Each experiment was performed in triplicate to ensure reproducibility of results.

Finally, to disrupt the interaction between importin α and ATF3, we performed a screen for small molecule inhibitors capable of blocking this protein‐protein interaction. A virtual screening was conducted against the binding interface of importin‐α and ATF3, specifically targeting the amino acid segments 100–200 and 400–550 of importin‐α, using a library of 2115 Food and Drug Administration (FDA)‐approved drugs (Figure 8A and Figure S8). Four candidate drug molecules were identified (Figure 8B), with the calculated binding free energies of their complexes with importin‐α as follows: importin‐α‑fda872, −26.93 kJ/mol; importin‐α‑fda1095, −47.730 kJ/mol; importin‐α‑fda1759, −28.91 kJ/mol; and importin‐α‑fda2098, −30.81 kJ/mol. Based on its most favorable total binding free energy among the candidates, fda1095 (Itraconazole) was selected for further experimental investigation (Figure 8C).

Next, we evaluated the effects of Itraconazole on the binding of Importin α and ATF3 in RAW264.7 macrophages. Immunoprecipitation confirmed that Itraconazole treatment reduced this SiO_2_‐induced the interaction between Importin α and ATF3 (Figure 8D). The nuclear accumulation of ATF3 induced by SiO_2_ was also reduced by Itraconazole stimulation (Figure 8E–G). In addition, treatment with Itraconazole protected macrophages against cellular senescence (Figure 8H,I) and mitochondrial dysfunction (Figure 8J–R) after SiO_2_ exposure. We further analyzed the effect of Itraconazole on fibroblast activation and found that culture supernatant derived from Itraconazole‐treated RAW264.7 macrophages significantly reduced SiO_2_‐induced fibroblast activation, contraction, and migration (Figure 8S–V). These data collectively demonstrate that the small‐molecule inhibitor Itraconazole inhibits the interaction between ATF3 and Importin α, thereby alleviating cellular senescence and fibroblast activation.

## 3 Discussion

In this study, we identified a mechanism by which SIRT6‐mediated ATF3 acetylation facilitates silica‐induced macrophage senescence and PF. Specifically, we demonstrated that ATF3 was significantly increased in silica‐induced senescent macrophages and fibrotic lungs. Through AlphaFold3, a deep learning‐based method for protein structure prediction, we confirmed that SIRT6 is a key regulator of acetylation modification of ATF3. Deacetylated ATF3 further promoted nuclear translocation and transcriptional activation of MGARP, leading to mitochondrial dysfunction, macrophage senescence, and PF. Furthermore, we specifically focused on the potential mechanisms underlying the increased nuclear translocation of ATF3. We showed that HSP70 and Importin α competitively bind to ATF3, affecting its nuclear transport. Both Importin α inhibitor and small molecule inhibitors targeting the binding of ATF3 and Importin α could reduce the nuclear translocation of ATF3, alleviate cellular senescence, and PF. Collectively, our findings reinforce the current insight of ATF3 as an important regulator of macrophage senescence in silicosis pathologies.

Silicosis is a chronic occupational lung disease due to long‐term exposure to silica dust, while physiological senescence of the organism does not appear to be its primary factor of onset [33, 34]. However, recent studies have shown that cellular senescence is closely related to the progression of silicosis. Cellular senescence is considered one of the hallmarks of aging, which refers to irreversible cell cycle arrest [35, 36]. Studies conducted by us have identified that a variety of different cell types, including senescent fibroblasts, epithelial cells, and endothelial cells, are playing a key role in silicosis [37, 38, 39]. Furthermore, a recent study found that senescence‐associated proteins of P16 and P21 were upregulated in RAW264.7 cells after SiO_2_ treatment, suggesting macrophage senescence may be involved in the development of silicosis [40]. Therefore, the mechanism of SiO_2_‐induced macrophage senescence warrants further investigation.

It is worth noting that the polarization state of senescent macrophages exhibits tissue heterogeneity and is not dominated by a single M1 or M2 phenotype; rather, it varies depending on the tissue environment, showing two opposing trends or a mixed M1/M2 phenotype [20]. For example, the frequency of M2‐like macrophages increases in aged human skeletal muscle, and similar observations have been made in mice [41, 42]. In aged mice, immunosuppressive M2 macrophages are significantly increased in the muscle [43]. In contrast, certain tissues show enhanced M1‐like (pro‐inflammatory) polarization during aging [44, 45]. For instance, the expression of the M1 marker CD86 is elevated in macrophages derived from peripheral blood monocytes of aged individuals, indicating a pro‐inflammatory state [46]. In aged mice, macrophages in adipose tissue and the liver are more inclined toward the M1 phenotype, exacerbating inflammation and liver injury [44, 45, 47]. These findings suggest that the polarization of aged macrophages is tissue‐dependent, with an M2 shift in most tissues but sustained or enhanced M1 polarization in some tissues (e.g., adipose tissue and liver), reflecting the coexistence of immune regulation and inflammation during aging. However, in our present results, we did not observe significant changes in the classical M1 or M2 markers during SiO_2_− or etoposide‐induced senescence (Figure S1M–P). This discrepancy may be attributed to several factors. First, unlike replicative senescence models, we established a premature senescence model of RAW264.7 macrophages (passage number < 10) induced by SiO_2_ or etoposide, which may affect their polarization phenotype. Second, the treatment duration (24 h) and concentrations (150 µg/mL for SiO_2_ and 10 µm for etoposide) used in our study may not have been sufficient to significantly impact the polarization state of RAW264.7 macrophages. Future studies using different treatment durations and concentrations could help further explore polarization changes in senescent macrophages. Third, no specific growth factors known to promote macrophage polarization were added to our culture medium, which may also account for the lack of a notable polarization shift. More importantly, our results provide preliminary evidence that SASP secreted by senescent macrophages, especially SPP1, drives functional changes in the lung tissue microenvironment and ultimately promotes PF progression, independent of classical macrophage polarization shifts.

In this work, based on multi‐omics data and multiple verification experiments, we demonstrated that SiO_2_ could induce macrophage senescence, which was accompanied by an increase in ATF3 expression. ATF3 is a transcription factor of the ATF/cAMP‐responsive element‐binding protein (CREB) family, which originally acted as an important mediator of adaptive response [48]. The expression of ATF3 is regulated by multiple cellular stresses, including DNA damage, ROS, and endoplasmic reticulum stress, playing a crucial role in various pathophysiological processes such as cancer, inflammation, and fibrosis [49, 50]. ATF3 has recently been found to regulate autophagy and contribute to the progression of PF [51]. It is noteworthy that ATF3 expression increased in lung epithelial cells with aged lungs, which negatively regulated PINK1 transcription and disrupted mitochondrial homeostasis [52]. However, the role of ATF3 in cellular senescence among different diseases and cell types is controversial. In certain models, ATF3 protects against vascular smooth muscle cell (VSMC) senescence and atherosclerosis (AS) by orchestrating autophagy through the ATF3‐ATG7 amplification loop [53]. The role of ATF3 in senescent macrophages and the development of silicosis has not yet been studied. We found that ATF3 was upregulated in SiO_2_‐induced senescent macrophages, and performed RNA transcriptome sequencing using control, SiO_2_, and SiO_2_ plus Atf3 siRNA‐treated RAW264.7 cells to explore the downstream targets of ATF3. Our findings preliminarily identified MGARP, an essential protein for maintaining mitochondrial structural integrity and regulating mitochondrial dynamics, balance, and functional homeostasis [54], as a downstream target of ATF3. Importantly, knocking down ATF3 in macrophages alleviated mitochondrial dysfunction and cellular senescence, thereby relieving silica‐induced PF. Taken together, our results provided strong evidence that increased expression of ATF3 contributed to macrophage senescence and PF.

Epigenetic alterations, such as methylation, acetylation, and phosphorylation, are known to drive the phenotypes of aging and cellular senescence [55]. In the present study, we observed that SiO_2_ significantly reduced the level of ATF3 acetylation in senescent macrophages. Acetylation is a general post‐translational modification of both histone and non‐histone proteins, thereby regulating protein function [56, 57, 58]. The acetylation level of transcription factors can affect their protein stability, transcriptional activity, subcellular localization, and interactions with other proteins [59, 60]. For example, the transcriptional level of forkhead box m1 (FOXM1) was related to H3K27ac modification [61]. Moreover, the deacetylation of nuclear factor erythroid 2‐related factor 2 (NRF2), regulated by SIRT2, could decrease nuclear translocation of NRF2 and its subsequent transcriptional activation capability [62]. Consistent with these findings, our study demonstrated that the deacetylation of ATF3 mediated by SIRT6 increased nuclear transport of ATF3, and thereby transcriptional activation of its downstream target gene MGARP. Furthermore, it is noteworthy that knockdown of SIRT6 or mutation of the ATF3 acetylation site significantly diminished ATF3 nuclear translocation and subsequent senescence‐related phenotypes.

Another interesting finding was that we further delved into the potential mechanisms influencing the nuclear import of ATF3. Under normal conditions, HSP70 binds to ATF3 and transports it to the lysosome for degradation. However, the expression of HSP70 was decreased after SiO_2_ treatment, and the reduced HSP70 impaired lysosomal function, leading to a decrease in the degradation of ATF3. HSP70 is an important chaperone with a substrate‐binding domain, which assists protein folding, transport, and degradation [63]. Previous research reported that HSP70 selectively recognized Parkinsonism‐associated peptidase 7 (PARK7) via KFERQ‐like motifs, facilitating its translocation to the lysosomal membrane through chaperone‐mediated autophagy [64]. Additionally, we found that ATF3 possessed a functional NLS sequence, in which the nuclear transport protein importin α could bind to ATF3 and facilitate the nuclear transport of ATF3. Usually, the importin‐α/β1 complex mediates nuclear import by recognizing and binding the NLS via importin‐α, followed by importin‐β1‐driven translocation through the nuclear pore [31]. In this study, we further identified that HSP70 and Importin α competitively bind with ATF3, thereby regulating ATF3 nuclear entry.

Based on the predicted binding sites, we performed virtual screening to identify small‐molecule inhibitors that may disrupt the interaction between Importin α and ATF3, using the FDA database in ZINC. Virtual screening is now widely used to discover small‐molecule drugs targeting specific proteins. For example, virtual screening against the family with sequence similarity 171 member A2 (FAM171A2) protein structure has identified bescitinib as a specific therapeutic molecule for Parkinson's disease [65]. Similarly, this approach led to the discovery of celastrol, which induces ferroptosis in pancreatic cancer cells by disrupting the Sorcin‐PAX5 interaction [66]. Additionally, bufalin has been successfully characterized as a molecular glue degrader targeting estrogen receptor α through virtual screening [67]. In our present study, the candidate small molecule inhibitor should exhibit high binding affinity for the same target site, thereby competitively inhibiting the interaction between Importin α and ATF3. We identified Itraconazole, which interfered with the amino acids critical for the Importin α‐ATF3 interaction and demonstrated Itraconazole's ability to prevent ATF3 nuclear entry and macrophage senescence. Itraconazole is a widely used antifungal drug and approved by the FDA for clinical practice [68]. Recently, several studies have indicated that itraconazole, in addition to its established antifungal activity, plays significant roles in other diseases such as cancer and fibrosis‐related disorders [69, 70]. In the present study, our structure‐based approach enabled the discovery of itraconazole as a therapeutic compound that modulates the nuclear translocation of ATF3.

Our findings highlight how deacetylated ATF3 facilitates macrophage senescence and silicosis, and show that targeting ATF3 and its nuclear transport provides a further potential strategy for treating cellular senescence and fibrotic diseases.

## 4 Experiment Section

### 4.1 Ethics Statement

Male C57BL/6 mice (6‐8 weeks) were housed under pathogen‐free conditions with food and water ad libitum. All experimental procedures were conducted in accordance with institutional and regulatory guidelines approved by the Animal Ethics Committee of Nantong University (Approval No. P20250418‐008). Human lung tissue sections were collected from patients with normal and silicosis in Wuxi No 8 People's Hospital (ethical approval number: 2025‐Y‐2). Informed consent forms were signed by donors or their families before sample collection. The experiments were approved by the Human Assurance Committee of Wuxi No 8 People's Hospital (Wuxi, China).

### 4.2 Animal Models and Treatments

Male C57BL/6 mice were acclimatized for 1 week under specific pathogen‐free (SPF) conditions. Pulmonary fibrosis was induced by single intratracheal instillation of 50 µL SiO_2_ suspension (50 mg/mL) under sodium pentobarbital anesthesia (1%, i.p.), with controls receiving 50 µL saline. For importin α inhibitor intervention, Ivermectin (MedChemExpress, HY‐15310; 3 mg/kg/day, i.p.) or vehicle (DMSO) was administered for 4 weeks starting 24 h post‐SiO_2_ exposure. For ATF3 knockdown, ATF3‐shRNA adenovirus (GeneChem, ≈ 6.28 × 10^10^ vg mouse^−^^1^) was delivered via intratracheal injection 4 weeks before SiO_2_ challenge. Mice were monitored daily with weight recorded; at endpoint (Day 28 post‐SiO_2_), lungs were harvested under anesthesia and stored at −80°C.

### 4.3 Cell Culture and Treatment

RAW264.7 and NIH/3T3 cells (ATCC) were cultured in DMEM (Gibco, Grand Island, USA) supplemented with 10% FBS (Gibco, 10270‐106) and 1% penicillin–streptomycin at 37°C in a humidified incubator with 5% CO_2_. For gene silencing, siAtf3, siSirt6, siHspa8, or siMgarp (50 nM) were introduced using a siRNA transfection reagent (Ribobio, C10511‐05), whereas ATF3 Q/R acetylation mutant plasmids (2.5 µg/well) were delivered with a plasmid transfection reagent (Beyotime, C0533); transfections were performed 48 h before subsequent treatments. The sequences of Atf3, Sirt6, Hspa8, and Mgarp siRNAs are listed in Table S1. RAW264.7 cells were then exposed to SiO_2_ (150 µg/mL, 24 h), with co‐treatment of etoposide (10 µm), nicotinamide mononucleotide (NMN, 400 µm), ivermectin (10 µm), itraconazole (1.5 µm), trichostatin A (TSA, 0.5 µm; inhibitor of histone deacetylase classes I, II, and IV), or nicotinamide (NAM, 100 µM; inhibitor of the SIRT family deacetylases) as indicated. To inhibit protein palmitoylation, SiO_2_‐treated RAW264.7 cells were additionally incubated with the palmitoylation inhibitor 2‐bromopalmitate (2‐BP, 50 µm) for 3 h before harvest for analysis of ATF3 nuclear and cytoplasmic protein levels. For direct treatment of fibroblasts with SPP1: SPP1 (50 nm, Biodragon) was added directly to NIH/3T3 cells in the presence or absence of a CD44‐neutralizing antibody (10 µg/mL, Bio X Cell). For indirect treatment of fibroblasts with SPP1: SPP1 was first added to RAW264.7 macrophages. After 24 h of treatment, the conditioned medium from RAW264.7 macrophages was collected and then applied to NIH/3T3 cells, again in the presence or absence of the CD44‐neutralizing antibody added to the NIH/3T3 cells.

### 4.4 Lung Tissue and Histology

Right lung tissues were fixed in 4% paraformaldehyde (PFA), paraffin‐embedded, and sectioned at 4 µm. Sections underwent hematoxylin and eosin (H&E) staining for histopathological evaluation; Masson's trichrome and Sirius red staining for collagen deposition quantification. IHC analysis as follows: After deparaffinization, antigen retrieval, and blocking with 5% goat serum, sections were incubated overnight at 4°C with primary antibodies (Table S2), followed by HRP‐conjugated secondary antibody incubation, DAB (Sigma–Aldrich) development, and hematoxylin counterstaining. All stained sections were dehydrated, cleared, and mounted for microscopic assessment.

### 4.5 Hydroxyproline Determination

Lung tissue (20 mg) was hydrolyzed (97°C, 20 min), pH‐adjusted, and diluted to 5 mL. After activated charcoal treatment and centrifugation (3500 g, 10 min), hydroxyproline was quantified in supernatants using a commercial kit (Jiancheng Bioengineering, China; Cat# A030‐2‐1) following the manufacturer's protocol.

### 4.6 Senescence‐Associated β‐Galactosidase Assay (SA‐β‐gal)

Cells or cryosections were fixed in β‐galactosidase fixative (15 min), washed with PBS, and incubated with staining solution at 37°C (24–48 h). SA‐β‐gal‐positive cells were quantitatively assessed by bright‐field microscopy.

### 4.7 Immunofluorescence

Paraffin‐embedded lung sections were deparaffinized, rehydrated, and subjected to citrate‐based antigen retrieval. After endogenous peroxidase quenching and blocking with 5% goat serum, sections underwent sequential multiplex staining using a TSA kit (AFIHC023, Aifang Bio, China): First incubated overnight at 4°C with primary antibody, followed by HRP‐polymer secondary and red fluorophore development; subsequently stripped, reblocked, and incubated with another primary antibody developed with green fluorophore. Nuclei were counterstained with DAPI, and mounted sections were imaged by confocal microscopy (Nikon). For the immunofluorescence‐based localization analysis, specifically, ATF3 fluorescence was quantified in ImageJ. Nuclear regions were defined using DAPI staining, whole‐cell regions were manually outlined, and the cytoplasmic signal was calculated by subtracting the nuclear region from the whole‐cell region. After background subtraction, the nuclear/cytoplasmic fluorescence ratio was calculated for each cell to assess ATF3 nuclear translocation. At least 30 cells per group from 3 random fields were analyzed in three independent experiments.

### 4.8 Coimmunoprecipitation Assay

Protein lysates (1 mg) were immunoprecipitated with target antibody (1 µg; 4°C overnight), captured by Protein A/G beads (3 h at 4°C), washed three times with PBS, and eluted in SDS sample buffer. Input samples were analyzed in parallel.

### 4.9 Western Blotting

Total protein was extracted using RIPA lysis buffer (Beyotime, #P1046, China) with protease inhibitors and quantified by BCA assay (Beyotime, #P0009, China). Proteins (30 µg/lane) were separated on 10% or 15% SDS‐PAGE gels, transferred to polyvinylidene difluoride (PVDF, Millipore Corporation, Billerica, #MA 01821, USA), and blocked with 5% skim milk. Membranes were incubated overnight at 4°C with primary antibodies (Table S2), followed by HRP‐conjugated secondary antibody (room temperature, 1 h). Protein bands were visualized using a Tanon‐5200 system (Tanon Company, Shanghai, China) and quantified via ImageJ with β‐actin normalization.

### 4.10 Real‐Time Quantitative PCR (qRT‐PCR) Analysis

Total RNA was extracted from lung tissues using TRIzol reagent (Takara, Japan), quantified by NanoDrop 8000 spectrophotometer (Thermo Fisher Scientific, USA), and reverse‐transcribed with Omniscript RT Kit (Takara, Japan) using Oligo dT primers. RT‐qPCR was performed with TB Green Premix (Takara, Japan) on a LightCycler 480 system (Roche, Switzerland). Gene expression was calculated via the 2^−ΔΔCt^ method, normalized to GAPDH. Primer sequences are provided in Table S3.

### 4.11 Chromatin Immunoprecipitation (ChIP)

To examine ATF3 binding to the MGARP promoter, ChIP assays were performed using the Smart‐ChIP ChIP Kit (Engibody, cat. no. ChIP‐1001) following the manufacturer's instructions. Briefly, cells were crosslinked with 1% formaldehyde at room temperature for 10 min, and the reaction was stopped by adding the supplied 10× stop solution. After two washes with ice‐cold PBS, cells were collected and incubated in Chromatin Extract Buffer on ice to release nuclei. Nuclear lysates were sonicated to fragment chromatin to an average size of approximately 200–500 bp, and insoluble material was removed by centrifugation. A small aliquot of sheared chromatin (1% of the total) was reserved as input, and the remaining chromatin was incubated overnight at 4°C with ChIP‐grade anti‐ATF3 antibody or normal isotype IgG, followed by capture with Protein A/G magnetic beads. Bead‐bound immune complexes were washed sequentially with ChIP Wash Buffers 1–4 and eluted with ChIP Elution Buffer prepared as recommended by the manufacturer. Crosslinks were reversed at elevated temperature in the presence of Proteinase K, followed by RNase A treatment and DNA purification using the kit DNA precipitation reagent and ethanol. The recovered DNA was dissolved in nuclease‐free water and subjected to ChIP–qPCR using the 2× real‐time PCR master mix included in the kit and primers spanning the MGARP promoter region containing the predicted ATF3‐binding motif; the specific primer sequences are listed in Table S4. ChIP enrichment was calculated after normalization to input DNA and expressed as fold enrichment relative to the IgG control.

### 4.12 Luciferase Reporter Assay

Luciferase reporter assay was conducted using the Double luciferase reporter gene detection kit (Yeasen Biotechnology, Shanghai). RAW264.7 macrophages were seeded into 96‐well plates and co‐transfected with Mgarp wild‐type plasmid or the binding mutant plasmid. After 48 h of incubation, the firefly and Renilla luciferase activities were examined with a dual‐luciferase reporter assay.

### 4.13 Transwell Migration Assay

NIH/3T3 cells in Transwell chambers (Costar, USA) were cultured for 24 h under experimental conditions. Cells were fixed with 4% PFA and stained with crystal violet (Beyotime, #C0121). Non‐migrated cells on the upper membrane surface were then removed by a cotton swab. Migrated cells on the lower surface were quantified by counting three random fields per well under light microscopy.

### 4.14 Collagen Gel Contraction Assay

NIH/3T3 cells were embedded in neutralized rat tail collagen I (1 mg/mL final; Hangzhou Xinyou Biotechnology) prepared in ice‐cold conditions. The mixture was immediately transferred to culture dishes, polymerized at room temperature for 20 min, and cultured in complete medium.

### 4.15 Senescent Macrophage‐Derived Conditioned Medium Preparation

For the preparation of senescent RAW264.7‐derived conditioned media, senescence of macrophages was induced with the treatments (SiO_2_ at 150 µg/mL for 24 h combined with NMN (400 µm), siRNA targeting ATF3/MGARP, ATF3 acetylation mutants (Q/R), Ivermectin (10 µm), or Itraconazole (1.5 µm)). Following treatment, cells were washed twice with PBS and cultured in serum‐free DMEM for 24 h. Supernatants were centrifuged to remove debris and immediately added to NIH/3T3 cells for 24 h.

### 4.16 Macrophage Phagocytosis Assay

Phagocytic capacity of alveolar macrophages was assessed using fluorescent microspheres (6 h co‐incubation). For the microsphere assay, internalized particles were quantified by fluorescence microscopy.

### 4.17 Mitochondrial Function Assay

RAW264.7 macrophages (induced by SiO_2_ at 150 µg/mL for 24 h combined with: itraconazole (1.5 µm), ATF3‐Q/R acetylation mutants, siAtf3, or siMgarp) were stained on coverslips with: MitoSOX Red (5 µm, Thermo #M36008) for mitochondrial ROS, NAO (1:1000 dilution, Thermo #A1372) for mitochondrial mass, JC‐1 (Beyotime #C2006) for membrane potential assessment (red/green ratio), and MitoTracker (200 nm, Beyotime #C1035) for mitochondrial mass detection. All probes were incubated in serum‐free medium at 37°C for 30 min, protected from light, washed with PBS, fixed with 4% PFA, and imaged by fluorescence microscopy.

### 4.18 CHX Assay

Cells were treated with 20 µm cycloheximide (Cell Signaling Technology, #2112) and harvested at 0, 3, 6, and 9 h post‐treatment. Whole‐cell lysates were prepared using RIPA buffer supplemented with protease inhibitor cocktail, followed by western blotting analysis to quantify protein degradation kinetics.

### 4.19 Nuclear‐Cytoplasmic Fractionation

Nuclear and cytoplasmic fractions were isolated using Beyotime Kit (#P0028): PBS‐washed cells were lysed in Reagent A + 1 mm PMSF, vortexed 5 sec, incubated on ice 15 min, mixed with Reagent B, centrifuged (12 000 g, 5 min, 4°C) to collect cytoplasmic supernatant; nuclear pellets were resuspended in Nuclear Reagent + 1 mM PMSF, vortexed intermittently (15–30 sec/2 min × 30 min on ice), centrifuged (12 000 g, 10 min, 4°C), with extracts stored at −80°C.

### 4.20 Transcriptome Data Analysis

RNA Sequencing of RAW264.7 macrophages and analysis as follows: RNA was extracted from triplicate samples of RAW264.7 macrophages treated with control (untreated), SiO_2_ (150 µg/mL, 24 h), or SiO_2_ + siAtf3 conditions; libraries were prepared using NEBNext Ultra II RNA Kit (NEB #E7770), sequenced on Illumina NovaSeq 6000 (PE150, 30M reads/sample), with raw reads aligned to Mus musculus genome (GRCm38) via STAR v2.7.10a, followed by differential expression analysis using DESeq2 v1.38.3 (FDR<0.05, |log2FC|>1) and senescence pathway assessment via GSEA (MSigDB Hallmarks).

RNA Sequencing of mouse lung tissues and analysis as follows: total RNA was extracted from lung tissues of control mice and those exposed to silica for 28 days, with three mice randomly selected per group. RNA quality was initially checked using agarose gel electrophoresis and a Nanodrop spectrophotometer, followed by assessment of RNA integrity numbers (RIN) on an Agilent 2100 Bioanalyzer. Sequencing libraries were prepared with the NEBNext UltraTM RNA Library Prep Kit for Illumina according to the manufacturer's protocol, then pooled and sequenced on an Illumina Novaseq 6000 platform. After sequencing data underwent quality control, clean reads were mapped to the mouse reference genome using Hisat2 (http://ccb.jhu.edu/software/hisat2). Transcript assembly, quantification, and differential expression analysis were performed with the Cufflinks software package (http://cole‐trapnell‐lab.github.io/cufflinks). Read counts per annotated gene were obtained using HTseq‐count (http://htseq.readthedocs.io/en/release_0.9.1), and FPKM (Fragments per Kilobase Million) values were calculated via custom scripts.

Single‐cell transcriptome sequencing data analysis was conducted as described previously. Single‐cell libraries were prepared using the Chromium Single Cell 3′ V3.1 Kit (10x Genomics) with 1000 cells/µL per channel. Sequencing was performed on an Illumina NovaSeq 6000 (PE150). Raw data were processed via Cell Ranger v6.1.1 to generate feature‐barcode matrices. After quality control (>200 genes/cell, <20% mitochondrial UMIs) and integration using Seurat v4.3, macrophage subclusters were identified through UMAP clustering. ATF3 expression dynamics were quantified across subclusters, and Pearson correlation analysis assessed its association with senescence markers Cdkn1a in macrophage populations. Public silicosis scRNA‐seq data (three silicosis patients and three healthy donors; GEO accession numbers: GSM8808455, GSM8778051, GSM8778048, GSM3660642, GSM3660643, GSM3660649) were acquired. IPF scRNA‐seq data (GSE128033) were processed using Seurat (v4.3.0). Count matrices were loaded, and a Seurat object was created per sample, retaining genes detected in ≥3 cells and cells with ≥200 detected genes.

### 4.21 LC‐MS/MS Analysis

ATF3‐immunoprecipitated proteins were digested on‐filter with trypsin (20 ng/µL, 16 h), desalted, and analyzed by LC‐MS/MS: Easy nLC 1200 with 60‐min gradient (5→100% B; B: 80% ACN/0.1% FA) coupled to Q‐Exactive HF (DDA mode; 120K/15K resolution, HCD 28% NCE). MaxQuant‐searched data (UniProt UP000000589, FDR<1%) identified HSPA8 with ≥2 unique peptides and >5‐fold enrichment versus IgG.

### 4.22 Molecular Docking and Dynamics Simulation

The crystal structure of ATF3 (PDB: 6NJS) and homology‐modeled MGARP (SWISS‐MODEL, UniProt Q8N5F7) were energy‐minimized using CHARMM36 force field. Protein‐protein docking was performed with HADDOCK v2.4 (default parameters), clustering the top 10 poses by interface RMSD. The optimal ATF3‐MGARP complex underwent 100 ns all‐atom MD simulation in AMBER20 with ff14SB force field, solvated in TIP3P water (0.15 m NaCl). Binding free energy was calculated via MM/PBSA (last 20 ns trajectory), with per‐residue decomposition identifying key interaction hotspots.

### 4.23 Data and Materials Availability

All data supporting the results of this study are presented within the Article, Supplementary Information, or the Source Data file. Sequencing data are available as follows: the human scRNA‐seq data generated in this study have been deposited in GEO under accession code GSE128033; publicly available silicosis scRNA‐seq data (three silicosis patients and three healthy donors) were accessed from GEO under codes GSM8808455, GSM8778051, GSM8778048, GSM3660642, GSM3660643, and GSM3660649. The spatial transcriptomic sequencing data related to this study have been deposited in the GEO database under the code GSE264233. Additional information is available from the corresponding author upon reasonable request.

### 4.24 Statistical Analyses

Statistical analyses were performed using GraphPad Prism 8, with data presented as mean ± SD; significant differences (*p*<0.05) were determined by unpaired Student's *t*‐test for comparisons between two groups or one‐way ANOVA followed by Tukey's multiple comparisons test for three or more groups, with n≥3 biological replicates in all experiments.

Further information on research design is available in the Supplementary Information.

## Conflicts of Interest

The authors declare no conflicts of interest.

## Supporting information




**Supporting File 1**: advs75782‐sup‐0001‐SuppMat.docx.

**Supporting File 2**: advs75782‐sup‐0002‐FigureS1‐S8.zip.

## Data Availability

The data that support the findings of this study are available in the supplementary material of this article.
