# Supplementary material for: SIRT6‐Mediated Deacetylation of ATF3 Promotes Silica‐Induced Lung Fibrosis by Enhancing its Nuclear Import via Binding to Importin α
Source: Adv Sci (Weinh). 2026 May 20;13(46):e75782. doi: 10.1002/advs.75782 (PMC13335902; doi:10.1002/advs.75782)
Supplement: Supplementary file 1 [file ADVS-13-e75782-s001.docx]

**Supporting Information**

**Supplementary Figures**

**Fig. S1. Increased macrophage senescence in silicosis human and murine lung tissues**

(A) Heat map representation of cellular senescence markers in bulk RNA-seq data from PBS and silica-exposure (28 days) mice lung tissues (n = 3 for each group, biological replicates). (B) UMAP visualization of cellular populations from scRNA-seq data of normal and silica-induced fibrotic human lungs. (C) UMAP plots show CDKN1A-positive cells of normal and silica-induced fibrotic human lungs (n = 3 for each group, biological replicates). (D) Interaction numbers and strength between the different subpopulations. (E) UMAP visualization of macrophages from PBS and silica-induced fibrotic mouse lungs (n = 3 for each group, biological replicates). (F) Bar graph representing the rate of CDKN1A-positive macrophages in normal and silica-induced fibrotic human lungs. (G-I) Mice body, lung weight, and lung coefficient of mouse from control and silica-treated groups, all data were expressed as the means ± SD of at least 3 independent experiments, with ^*^p < 0.05 and ^****^p < 0.0001 vs. the indicated group, and p values were from a 2-tailed unpaired Student’s t-test. (J) Quantification of hydroxyproline content in mouse lung tissues after silica treatment, all data were expressed as the means ± SD of at least 3 independent experiments, with ^*^p < 0.05 vs. the indicated group, and p values were from a 2-tailed unpaired Student’s t-test. (K) Typical images of P16 and P21 IHC staining in control and silica-treated mouse lung tissues, scale bar = 50 µm. Each experiment was performed in triplicate to ensure reproducibility of results. (L) Representative fluorescent images of phagocytosis of fluorescent microspheres by RAW264.7 macrophages at different time points. Each experiment was performed in triplicate to ensure reproducibility of results. (M-N) Western blot and quantitative analysis of CD86 and CD206 in SiO_2_-induced RAW264.7 macrophages. All data were expressed as the means ± SD of at least 3 independent experiments. (O-P) Western blot and quantitative analysis of CD86 and CD206 in Etoposide-induced RAW264.7 macrophages. All data were expressed as the means ± SD of at least 3 independent experiments. (Q-R) Western blot and quantitative analysis of cellular senescence markers (P16 and P21) in SiO_2_ or NMN-treated RAW264.7 macrophages. All data were expressed as the means ± SD of at least 3 independent experiments, with ^**^p < 0.01, ^***^p < 0.001, and ^****^p < 0.0001 vs. the indicated group, and p values were from a 1-way ANOVA post hoc test with Tukey’s correction. (S-T) SA-β-Gal staining and quantification in SiO_2_ or NMN-treated RAW264.7 macrophages. All data were expressed as the means ± SD of at least 3 independent experiments, with ^***^p < 0.001 vs. the indicated group, and p values were from a 1-way ANOVA post hoc test with Tukey’s correction.

**Fig. S2. ATF3 is up-regulated in senescent macrophages of human and murine silicotic lungs**

(A) scRNA-seq showing macrophage profiles in PBS and silica-treated mouse lung tissues (n = 2 for each group, biological replicates). (B) qRT-PCR analysis of Runx1 in silica-treated mouse lung tissues. All data were expressed as the means ± SD of at least 3 independent experiments. (C) qRT-PCR analysis of Cebpa in silica-treated mouse lung tissues. All data were expressed as the means ± SD of at least 3 independent experiments. (D) qRT-PCR analysis of Atf3 in silica-treated mouse lung tissues. All data were expressed as the means ± SD of at least 3 independent experiments, with ^*^p < 0.05 and ^***^p < 0.001 vs. the indicated group, and p values were from a 1-way ANOVA post hoc test with Tukey’s correction. (E) Western blot analysis of ATF3 in silica-treated mouse lung tissues (n = 3 for each group, biological replicates). Each experiment was performed in triplicate to ensure reproducibility of results. (F-I) qRT-PCR analysis of Atf3 in macrophages, endothelials, epithelials, and fibroblasts, respectively. All data were expressed as the means ± SD of at least 3 independent experiments, with ^*^p < 0.05, ^**^p < 0.01, and ^****^p < 0.0001 vs. the indicated group, and p values were from a 1-way ANOVA post hoc test with Tukey’s correction. (J) Representative results for coimmunostaining of P21 and ATF3 in control and silica-treated mouse lung sections. Green represents ATF3, red represents P21, and blue represents nuclear DNA staining by DAPI, scale bar = 100 µm. Each experiment was performed in triplicate to ensure reproducibility of results. (K) ATF3/P21 overlap coefficient from the lung sections of control and silica-treated mouse lung tissues; all data were expressed as the means ± SD of at least 3 independent experiments, with ^**^p < 0.01 vs. the indicated group, and p values were from a 2-tailed unpaired Student’s t-test. (L-M) Western blot and quantitative analysis of ATF3 in RAW264.7 macrophages after SiO_2_ treatment. All data were expressed as the means ± SD of at least 3 independent experiments, with ^*^p < 0.05 vs. the indicated group, and p values were from a 2-tailed unpaired Student’s t-test. (N) UMAP plots show ATF3-positive macrophages of scRNA-seq data from human lung tissues of normal and IPF (n = 7 for the normal group and n = 8 for the IPF group, biological replicates). (O) Bar graph representing the number of ATF3-positive macrophages in normal and IPF lung tissues. (P) The strength between the different subpopulations according to scRNA-seq data from human lung tissues of normal and IPF (n = 7 for the normal group and n = 8 for the IPF group, biological replicates). (Q) Typical images of P16 and P21 IHC staining in AAV9-NC and AAV9-Atf3 treated mouse lung tissues, scale bar = 40 µm. Each experiment was performed in triplicate to ensure reproducibility of results. (R) Representative image of lung appearance in mice after AAV9-NC and AAV9-Atf3 treatments. (S-U) Mice body, lung weight, and lung coefficient of mice from AAV9-NC and AAV9-Atf3-treated groups. All data were expressed as the means ± SD of at least 3 independent experiments, with ^*^p < 0.05 and ^**^p < 0.01 vs. the indicated group, and p values were from a 2-tailed unpaired Student’s t-test. (V) Quantification of hydroxyproline content in AAV9-NC and AAV9-Atf3-treated lung tissues. All data were expressed as the means ± SD of at least 3 independent experiments, with ^**^p < 0.01 vs. the indicated group, and p values were from a 2-tailed unpaired Student’s t-test. (W-X) Typical images of Col I and α-SMA IHC staining in AAV9-NC and AAV9-Atf3-treated mouse lung tissues, scale bar = 40 µm. Each experiment was performed in triplicate to ensure reproducibility of results.

**Fig. S3. SPP1-CD44 signaling pathway mediates the crosstalk of senescent macrophages and fibroblasts**

(A) Neighborhood enrichment analysis of IPF spatial transcriptome. (B) UMAP clustering aligned with spatial localization. (C) Multi-scale co-occurrence analysis of gene pairs. (D-F) Spatial expression of (D) COL1A1, (E) SPP1, and (F) CD44 on H&E-stained tissue (purple-low, yellow-high). Scale bar=500μm. (G) Representative micrographs of cell migration in the transwell migration assay after SPP1-CM and anti-CD44 antibody treatment. Each experiment was performed in triplicate to ensure reproducibility of results. (H) Fibroblast contraction was measured using the collagen gel-based contraction assay after SPP1-CM and anti-CD44 antibody treatment. Each experiment was performed in triplicate to ensure reproducibility of results. (I-J) Western blot and quantitative analysis of fibrotic markers (Col I and α-SMA) in NIH/3T3 cells after SPP1-CM and anti-CD44 antibody treatment; all data were expressed as the means ± SD of at least 3 independent experiments, with ^*^ p < 0.05 and ^****^p < 0.0001 vs. the indicated group, and p values were from a 1-way ANOVA post hoc test with Tukey’s correction.

**Fig. S4. Atf3 transcriptional regulation of Mgarp mediates mitochondrial damage, macrophage senescence, and pulmonary fibrosis**

(A) Scatter plot of ATF3 versus FIS1 co‑expression from scRNA-seq data of normal and silicotic human lung tissues (n = 3 for each group, biological replicates). (B) Scatter plot of ATF3 versus DRP1 co‑expression from scRNA-seq data of normal and silicotic human lung tissues (n = 3 for each group, biological replicates). (C) Scatter plot of Atf3 versus Fis1 co‑expression from scRNA-seq data of normal and silicotic mouse lung tissues (n = 2 for each group, biological replicates). (D) Scatter plot of Atf3 versus Drp1 co‑expression from scRNA-seq data of normal and silicotic mouse lung tissues (n = 2 for each group, biological replicates). (E) Schematic diagram of ATF3 and the MGARP promoter. (F) Predicted structure of the ATF3-MGARP promoter complex using AlphaFold3. (G) Schematic diagram of the strategy for constructing mutant sites based on the AlphaFold3-predicted ATF3-MGARP promoter interaction. (H) qRT-PCR analysis of Mgarp in RAW264.7 macrophages after SiO_2_ and siAtf3 treatment; all data were expressed as the means ± SD of at least 3 independent experiments, with ^*^p < 0.05 and ^**^p < 0.01 vs. the indicated group, and p values were from a 1-way ANOVA post hoc test with Tukey’s correction. (I-J) Western blot and quantitative analysis of MGARP in mouse lung tissues; all data were expressed as the means ± SD of at least 3 independent experiments, with ^**^p < 0.01 vs. the indicated group, and p values were from a 2-tailed unpaired Student’s t-test. (K-L) Western blot and quantitative analysis of mitochondrial damage markers (DRP1, FIS1, and OPA1) in RAW264.7 macrophages after SiO_2_ treatment; all data were expressed as the means ± SD of at least 3 independent experiments, with^*^ p < 0.05, ^**^ p < 0.01, ^***^ p < 0.001, and ^****^p < 0.0001 vs. the indicated group, and p values were from a 1-way ANOVA post hoc test with Tukey’s correction. (M) Representative results for coimmunostaining and quantitative analysis of F4/80 and OPA1 in mouse lung sections. Green represents F4/80, red represents OPA1, and blue represents nuclear DNA staining by DAPI, scale bar = 100 µm. Each experiment was performed in triplicate to ensure reproducibility of results. (N) The mean fluorescence intensity of OPA1 in F4/80^+^ cells. All data were expressed as the means ± SD of at least 3 independent experiments, with ^**^ p < 0.01 vs. the indicated group, and p values were from a 2-tailed unpaired Student’s t-test. (O) Representative results for coimmunostaining and quantitative analysis of F4/80 and DRP1 in mouse lung sections. Green represents F4/80, red represents DRP1, and blue represents nuclear DNA staining by DAPI, scale bar = 100 µm. Each experiment was performed in triplicate to ensure reproducibility of results. (P) The mean fluorescence intensity of DRP1 in F4/80^+^ cells. All data were expressed as the means ± SD of at least 3 independent experiments, with ^**^ p < 0.01 vs. the indicated group, and p values were from a 2-tailed unpaired Student’s t-test. (Q) Representative results for coimmunostaining and quantitative analysis of F4/80 and FIS1 in mouse lung sections. Green represents F4/80, red represents FIS1, and blue represents nuclear DNA staining by DAPI, scale bar = 100 µm. Each experiment was performed in triplicate to ensure reproducibility of results. (R) The mean fluorescence intensity of FIS1 in F4/80^+^ cells. All data were expressed as the means ± SD of at least 3 independent experiments, with ^*^ p < 0.05 vs. the indicated group, and p values were from a 2-tailed unpaired Student’s t-test. (S-T) Representative images of NAO staining and relative NAO fluorescence quantification in RAW264.7 macrophages after SiO_2_ and siAtf3 treatment, scale bar = 100 µm. Each experiment was performed in triplicate to ensure reproducibility of results. All data were expressed as the means ± SD of at least 3 independent experiments, with ^**^p < 0.01 vs. the indicated group, and p values were from a 1-way ANOVA post hoc test with Tukey’s correction. (U-V) Representative images of SOX staining and relative SOX fluorescence quantification in RAW264.7 macrophages after SiO_2_ and siAtf3 treatment, scale bar = 100 µm. Each experiment was performed in triplicate to ensure reproducibility of results. All data were expressed as the means ± SD of at least 3 independent experiments, with ^**^p < 0.01 vs. the indicated group, and p values were from a 1-way ANOVA post hoc test with Tukey’s correction. (W-X) Representative images of JC-1 staining and relative JC-1 fluorescence quantification in RAW264.7 macrophages after SiO_2_ and siAtf3 treatment, scale bar = 100 µm. Each experiment was performed in triplicate to ensure reproducibility of results. All data were expressed as the means ± SD of at least 3 independent experiments, with ^**^p < 0.01 vs. the indicated group, and p values were from a 1-way ANOVA post hoc test with Tukey’s correction. (Y) Representative images of MitoTracker staining in RAW264.7 macrophages after SiO_2_ and siAtf3 treatment, scale bar = 5 µm. Each experiment was performed in triplicate to ensure reproducibility of results. (Z-AA) Western blot and quantitative analysis of mitochondrial damage markers (OPA1, FIS1 and DRP1) anf MGARP in RAW264.7 macrophages after SiO_2_ and siAtf3 treatment. All data were expressed as the means ± SD of at least 3 independent experiments, with ^*^ p < 0.05 and ^****^p < 0.0001 vs. the indicated group, and p values were from a 1-way ANOVA post hoc test with Tukey’s correction.

**Fig. S5. SIRT6-mediated ATF3 deacetylation facilitates its nuclear transport in senescent macrophages**

(A) RAW264.7 macrophages were treated with or without TSA, then were immunoprecipitated with anti-ATF3 antibody, followed by western blot assay of immunocomplexes with the indicated antibody. Each experiment was performed in triplicate to ensure reproducibility of results. (B) RAW264.7 macrophages were treated with or without NAM, then were immunoprecipitated with anti-ATF3 antibody, followed by western blot assay of immunocomplexes with the indicated antibody. Each experiment was performed in triplicate to ensure reproducibility of results. (C-D) Western blot and quantitative analysis of cellular senescence markers (P16 and P21), SIRT6, and ATF3 in RAW264.7 macrophages after SiO_2_ and NAM treatment. All data were expressed as the means ± SD of at least 3 independent experiments, with ^*^ p < 0.05 and ^***^p < 0.001 vs. the indicated group, and p values were from a 1-way ANOVA post hoc test with Tukey’s correction. (E) Immunofluorescence staining of ATF3 in RAW264.7 macrophages after SiO_2_ and siSirt6 treatment. Green represents ATF3, blue represents nuclear DNA staining by DAPI, scale bar = 100 µm. Each experiment was performed in triplicate to ensure reproducibility of results. (F) ATF3 fluorescence intensity was measured after SiO_2_ and siSirt6 treatment. At least 30 cells per group from 3 random fields were analyzed in three independent experiments, and all data were expressed as the means ± SD, with ^*^p < 0.05 and ^**^p < 0.01 vs. the indicated group, and p values were from a 1-way ANOVA post hoc test with Tukey’s correction. (G) A schematic diagram of construct design with ATF3 acetylation sites. (H-I) Western blot and quantitative analysis of mitochondrial damage markers (OPA1, FIS1 and DRP1) and cellular senescence markers (P16 and P21) in RAW264.7 macrophages after K136/K139-2R and K136/K139-2Q treatment. All data were expressed as the means ± SD of at least 3 independent experiments, with ^*^ p < 0.05, ^**^p < 0.01 and ^****^p < 0.001 vs. the indicated group, and p values were from a 1-way ANOVA post hoc test with Tukey’s correction.

**Fig. S6. Nuclear transport protein importin α mediates the nuclear transport of ATF3**

(A) Representative images of SA-β-Gal staining in different groups of RAW264.7 macrophages after SiO_2_ and Ivermectin treatment. Each experiment was performed in triplicate to ensure reproducibility of results. (B) Representative image of lung appearance in mice after silica and Ivermectin treatment. (C-E) Mice body, lung weight, and lung coefficient of mice after silica and Ivermectin treatment; all data were expressed as the means ± SD of at least 3 independent experiments, with ^*^p < 0.05 vs. the indicated group, and p values were from a 2-tailed unpaired Student’s t-test. (F) Quantification of hydroxyproline content in mouse lung tissues after silica and Ivermectin treatment; all data were expressed as the means ± SD of at least 3 independent experiments, with ^****^p < 0.0001 vs. the indicated group, and p values were from a 2-tailed unpaired Student’s t-test. (G) Typical images of α-SMA and Col I IHC staining in silica and Ivermectin-treated mouse lung tissues, scale bar = 40 µm. Each experiment was performed in triplicate to ensure reproducibility of results. (H) Typical images of P16 and P21 IHC staining in silica and Ivermectin-treated mouse lung tissues, scale bar = 40 µm. Each experiment was performed in triplicate to ensure reproducibility of results.

**Fig. S7. HSP70 impairs the nuclear import of ATF3 through competitive binding with Importin α**

(A-B) Western blot and quantitative analysis of HSP70 in RAW264.7 macrophages after siHspa8 treatment; all data were expressed as the means ± SD of at least 3 independent experiments, with ^*^ p < 0.05 vs. the indicated group, and p values were from a 2-tailed unpaired Student’s t-test. (C-D) Western blot and quantitative analysis of HSP70 in RAW264.7 macrophages after SiO_2_ treatment; all data were expressed as the means ± SD of at least 3 independent experiments, with ^*^ p < 0.05 vs. the indicated group, and p values were from a 2-tailed unpaired Student’s t-test. (E-F) CHX assay and quantitative analysis were performed to detect ATF3 expression. The graph below illustrates the statistical analysis of the remaining ATF3. Each experiment was performed in triplicate to ensure reproducibility of results. (G) Immunofluorescence staining of LAMP1 in RAW264.7 macrophages treated with siHspa8. Green represents ATF3, red represents LAMP1, and blue represents nuclear DNA staining by DAPI, scale bar = 5 µm. Each experiment was performed in triplicate to ensure reproducibility of results. (H) ATF3/LAMP1 overlap coefficient from the lung sections of silica and siHspa8-treated groups, all data were expressed as the means ± SD of at least 3 independent experiments, with ^**^p < 0.01 vs. the indicated group, and p values were from a 2-tailed unpaired Student’s t-test. (I-J) Western blot and quantitative analysis of LAMP1 in RAW264.7 macrophages after siHspa8 treatment; all data were expressed as the means ± SD of at least 3 independent experiments, with ^*^ p < 0.05 vs. the indicated group, and p values were from a 2-tailed unpaired Student’s t-test. (K) The HDOCK Confidence of ATF3-HSP70 and ATF3-importin α complex-HSP70, all data were expressed as the means ± SD of at least 3 independent experiments, with ^*^ p < 0.05 vs. the indicated group, and p values were from a 2-tailed unpaired Student’s t-test.

**Fig. S8. Itraconazole-mediated inhibition of the ATF3-Importin α interaction blocks ATF3 nuclear entry**

(A-D) Schematic diagram of Itraconazole inhibiting ATF3 binding to Importin α at different domains. (E) The root mean square deviation (RMSD) was calculated to measure the stability and flexibility of the protein and compound. Each data point was performed in triplicate to ensure reproducibility of results. (F) The number of hydrogen bonds between small molecules and their target proteins using the molecular dynamics simulation. Each data point was performed in triplicate to ensure reproducibility of results. (G) Used Gromacs 2025 for molecular dynamics simulations. Force field parameters were obtained using the Gromacs pdb2gmx tool and the AutoFF webpage. Each data point was performed in triplicate to ensure reproducibility of results.

**Supplementary Tables**

**Table S1. Sequences of Atf3, Sirt6, Hspa8 and Mgarp siRNA**

| **Gene Name** | **sense** | **antisense** |
| --- | --- | --- |
| Atf3 (mouse) | CACCCTTIGUCAAGGAAGATT | UCUTCCUUGACAAAGGGUGTT |
| Sirt6 (mouse) | GCUACGUGGAUGAGGUGAUTT | AUCACCUCAUCCACGUAGCTT |
| Hspa8 (mouse) | CCAGGCCAGUAUUGAGAUUTT | AAUCUCAAUACUGGCCUGGTT |
| Mgarp (mouse) | GGUGGAUAUUACACUTACATT | UGUAAGUGUAAUAUCCACCTT |

**Table S2. Antibodies Utilized in This Study**

| **Antibodies.** | **Source** | **Identifier** | **Dilution ratio** |
| --- | --- | --- | --- |
| **Fibronectin** | Abcam | ab45688 | 1:1000 for WB |
| **Collagen Type I** | proteintech | 14695 | 1:1000 for WB |
| **α-SMA** | Abways | CY5295 | 1:1000 for WB, 1:500 for IHC |
| **P16** | proteintech | 10883 | 1:1000 for WB, 1:1000 for IHC |
| **P21** | proteintech | 10355 | 1:1000 for WB, 1:500 for IHC, 1:500 for IF |
| **CD86** | abways | CY5238 | 1:1000 for WB |
| **CD206** | santa cruz | sc58986 | 1:1000 for WB |
| **ATF3** | abcam | ab207434 | 1:1000 for WB, 1:50 for IP |
| **ATF3** | Immunoway | YT0387 | 1:1000 for WB, 1:500 for IHC, 1:500 for IF |
| **DRP1** | proteintech | 12957 | 1:2000 for WB |
| **FIS1** | proteintech | 10956 | 1:2000 for WB |
| **MFN1** | proteintech | 13798 | 1:1000 for WB |
| **MFN2** | proteintech | 12186 | 1:5000 for WB |
| **OPA1** | proteintech | 27733 | 1:2000 for WB |
| **LAminA/C** | proteintech | 10298 | 1:5000 for WB |
| **β-Tublin** | proteintech | 10068 | 1:2000 for WB |
| **Acetylated-lysine** | Cell Signaling | 9441 | 1:1000 for WB |
| **Sirt6** | proteintech | 13572 | 1:1000 for WB, 1:50 for IP |
| **importin α1** | Immunoway | YT5691 | 1:1000 for WB, 1:50 for IP |
| **MGARP** | proteintech | 22569 | 1:2000 for WB |
| **F4/80** | proteintech | 28463 | 1:500 for IF |
| **Pan Methylated Lysine** | Immunoway | YM3429 | 1:2000 for WB |
| **Phospho-Tyrosine** | Immunoway | YM8867 | 1:2000 for WB |
| **Phospho-Serine/Threonine** | Immunoway | YM9299 | 1:2000 for WB |
| **Ubiquitin** | Cell Signaling | 3933 | 1:2000 for WB |
| **SUMO2-3** | proteintech | 10947 | 1:2000 for WB |
| **β-actin** | Immunoway | YM3028 | 1:20000 for WB |
| **GAPDH** | proteintech | 10494 | 1:20000 for WB |

**Table S3. Primer Sequence for RT-qPCR**

| **Gene Name** | **Forward primer (5'-3')** | **Reverse primer (3'-5')** |
| --- | --- | --- |
| Atf3 (Mus) | GGTGGAAAGTGCCCCTCAAA | GTCCATCCTCTGTTGGCACA |
| Runx1 (Mus) | CACGCCAGTTCCCTACTCTG | AGGTAGGTGTGGTAGCGAGA |
| Cebpa (Mus) | CGGTGGACAAGAACAGCAAC | ACGTTGCGTTGTTTGGCTTT |
| P21 (Mus) | CGAGAACGGTGGAACTTTG | CCAGGGCTCAGGTAGACCTT |
| P16 (Mus) | GGTCGCAGGTTCTTGGTC | CTCGCTGTCCTGGGTCTC |
| Mgarp (Mus) | CCTGGTGGAGACTACCGAGA | TGGGGTGATGCTGTTTCCTC |
| Sirt6 (Mus) | CCCCAAGTTTGACACCACCT | TCATCAGCGAGCATCAGGTC |
| Gapdh (Mus) | AAGAAGGTGGTGAAGCAGG | GAAGGTGGAAGAGTGGGAGT |

**Table S4. Primer Sequence for ChIP-qPCR**

| **Primer name** | **Forward (5’-3’)** | **Reverse (5’-3’)** |
| --- | --- | --- |
| Mgarp-CHIP | GGATAGGTAGGTGGACGTGG | GAAGCCGGGGGTTTATCACC |

**Table S5. Top 20 genes with highest Moran’s I values in spatial transcriptomic analysis of human IPF lung tissue**

| **Gene Name** | **Moran’s I** | **pval_norm** |
| --- | --- | --- |
| APOC1 | 0.527103 | 0 |
| MT-ND1 | 0.4884 | 0 |
| COL1A2 | 0.480224 | 0 |
| MT-ATP6 | 0.476377 | 0 |
| MGP | 0.475806 | 0 |
| SCGB1A1 | 0.475349 | 0 |
| MT-CO2 | 0.469324 | 0 |
| FTL | 0.455595 | 0 |
| CCDC80 | 0.454309 | 0 |
| IGFBP4 | 0.447116 | 0 |
| MMP9 | 0.445649 | 0 |
| APOE | 0.438981 | 0 |
| CST3 | 0.437195 | 0 |
| MT-ND4L | 0.436567 | 0 |
| COL1A1 | 0.429308 | 0 |
| BPIFB1 | 0.426549 | 0 |
| SPARC | 0.425697 | 0 |
| LIPA | 0.425181 | 0 |
| MT-CO3 | 0.422358 | 0 |
| SPP1 | 0.42184 | 0 |

**Notes:** Moran’s I statistic was employed to evaluate global spatial autocorrelation of gene expression, determining whether expression patterns are spatially clustered, dispersed, or randomly distributed across the tissue coordinates. Values range from -1 (perfect dispersion) to +1 (perfect clustering), with 0 indicating spatial randomness. This table presents the 20 genes exhibiting the strongest spatial aggregation (highest Moran's I) in the IPF microenvironment, serving as candidates for spatial markers and region-specific transcriptional niches.
