# Supplementary figures and images for: SIRT6‐Mediated Deacetylation of ATF3 Promotes Silica‐Induced Lung Fibrosis by Enhancing its Nuclear Import via Binding to Importin α
Source: Adv Sci (Weinh). 2026 May 20:e75782. Online ahead of print. doi: 10.1002/advs.75782 (PMC13335902; doi:10.1002/advs.75782)

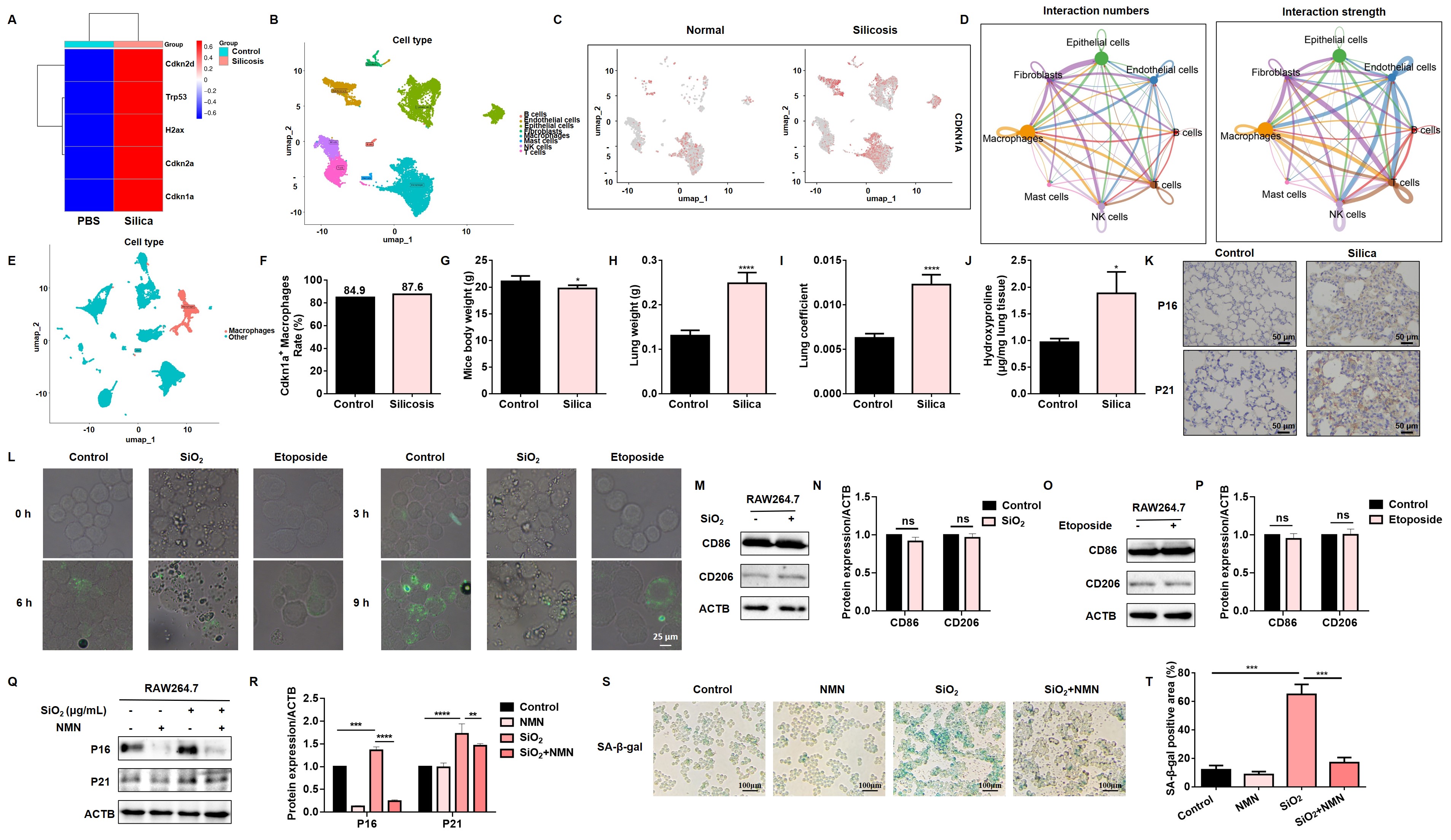

Supplement: Supplementary file 2 — Supporting File 2: advs75782‐sup‐0002‐FigureS1‐S8.zip. [file ADVS-9999-e75782-s002.zip › Figure S1.jpg]

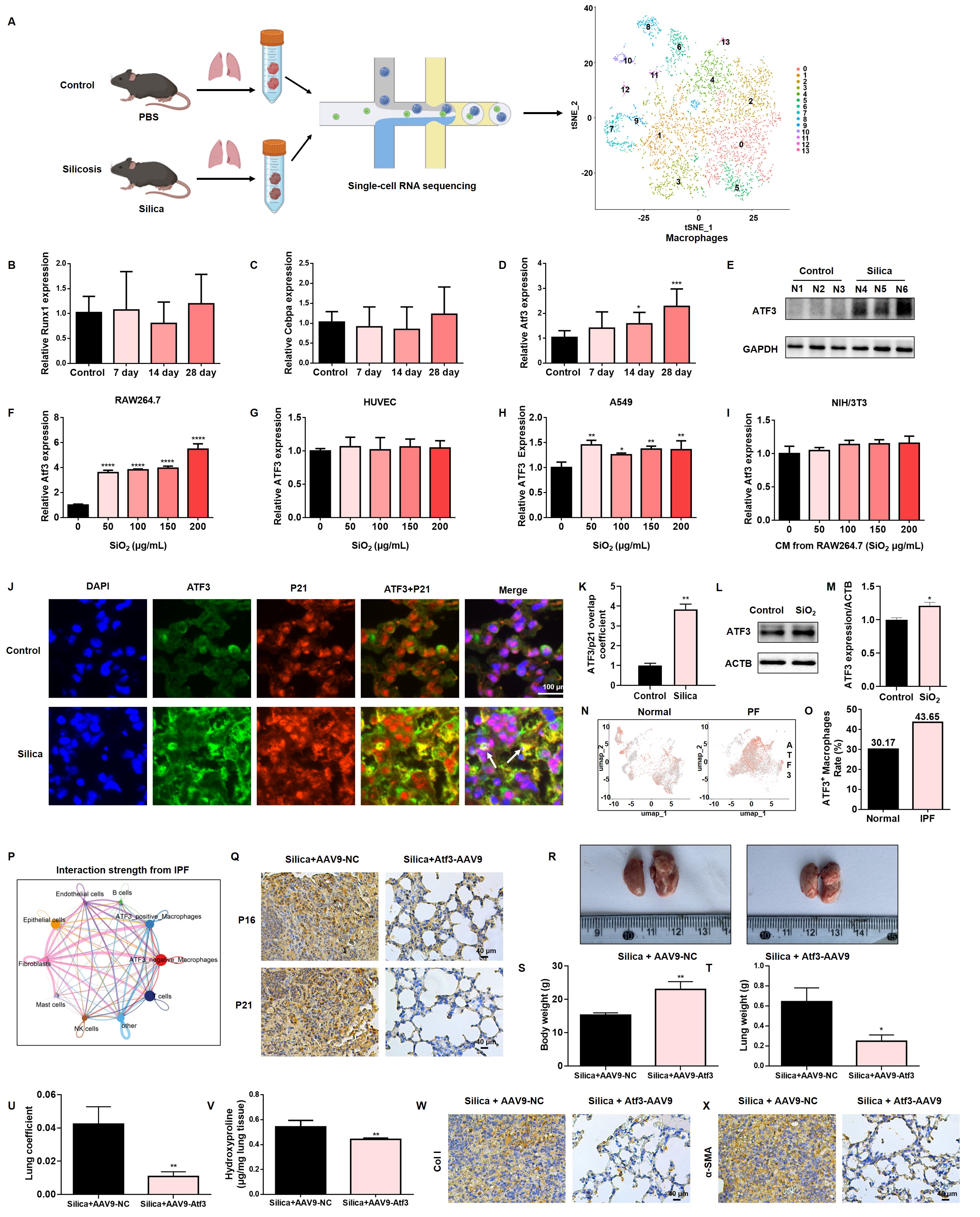

Supplement: Supplementary file 2 — Supporting File 2: advs75782‐sup‐0002‐FigureS1‐S8.zip. [file ADVS-9999-e75782-s002.zip › Figure S2.jpg]

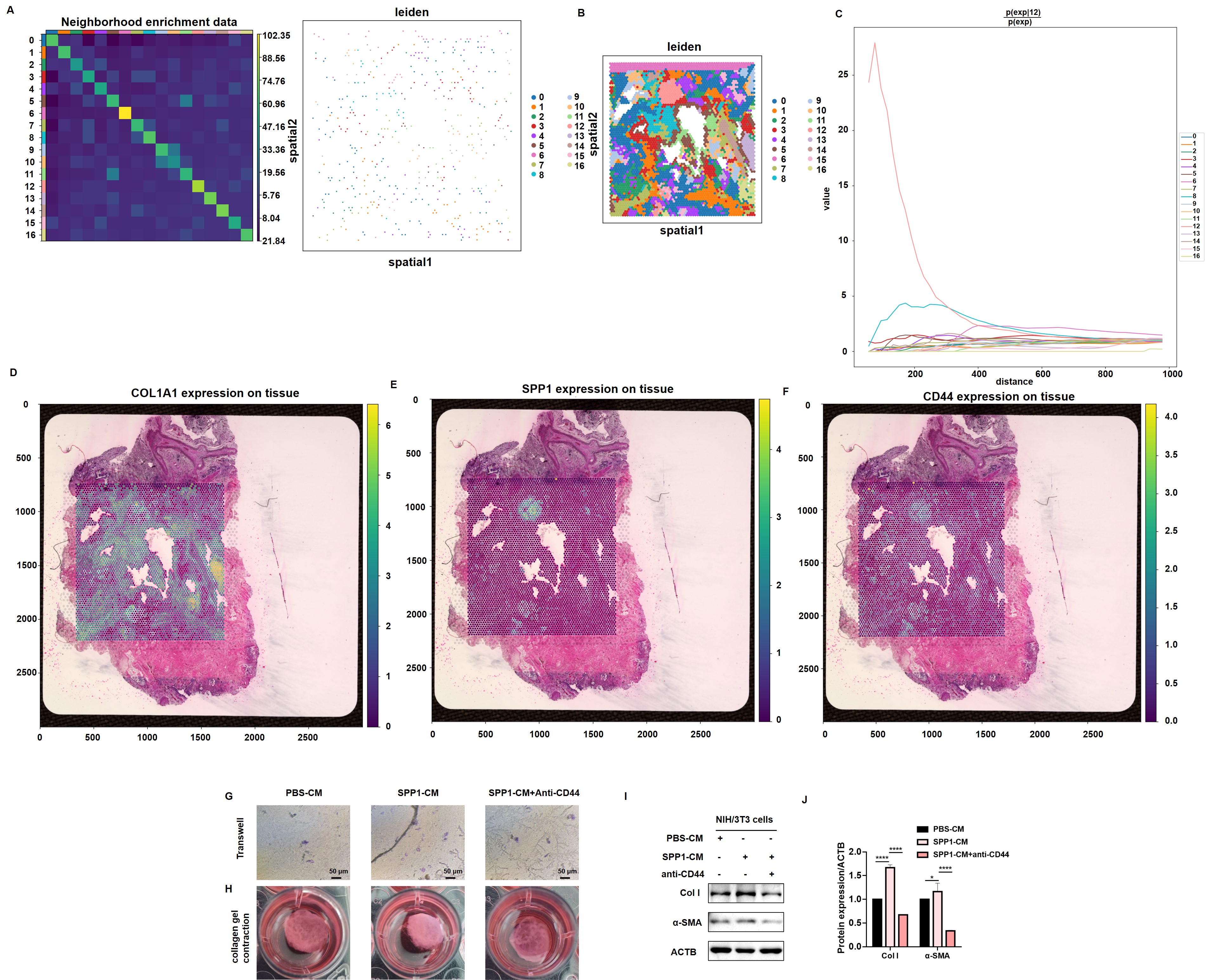

Supplement: Supplementary file 2 — Supporting File 2: advs75782‐sup‐0002‐FigureS1‐S8.zip. [file ADVS-9999-e75782-s002.zip › Figure S3.jpg]

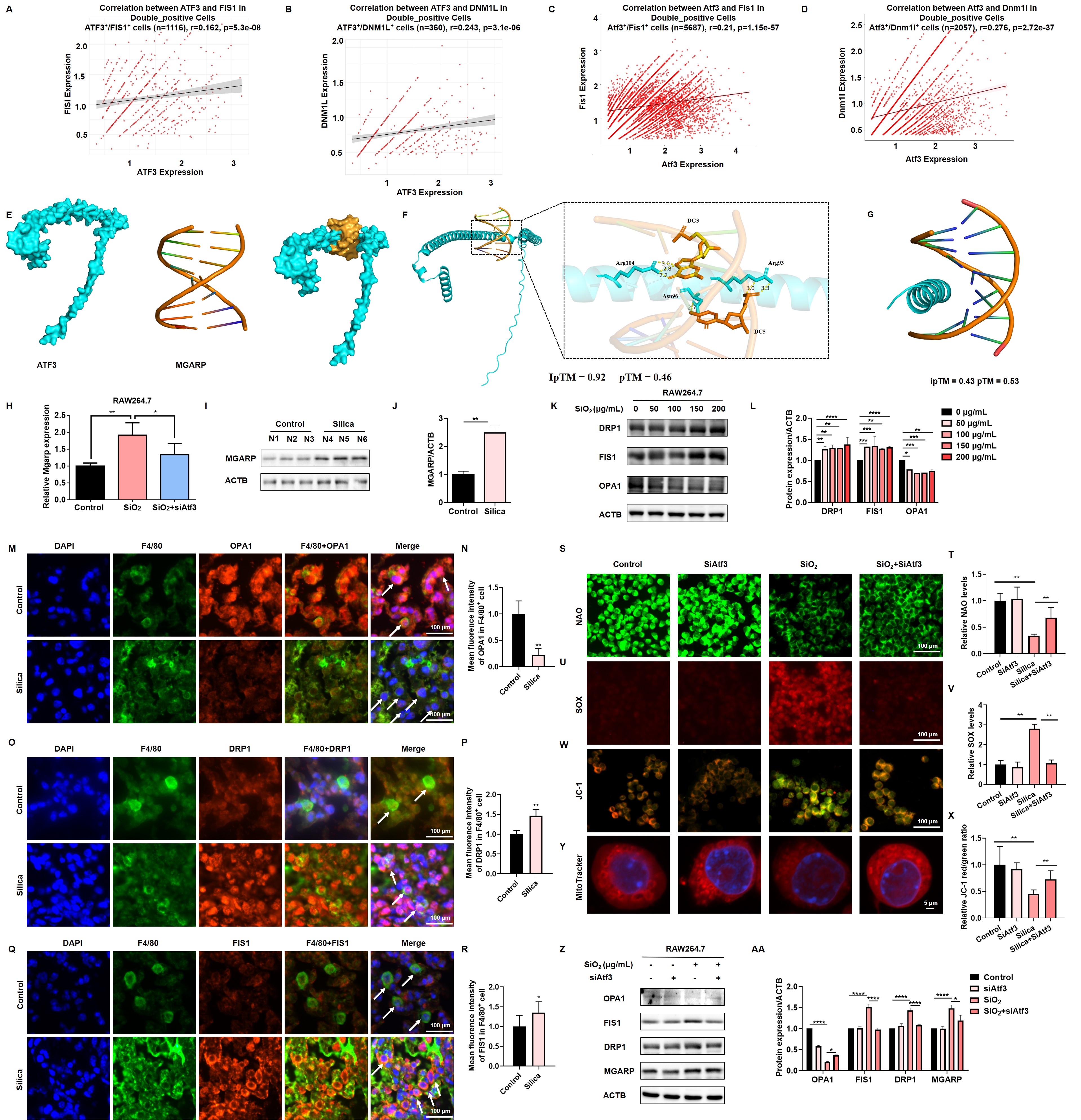

Supplement: Supplementary file 2 — Supporting File 2: advs75782‐sup‐0002‐FigureS1‐S8.zip. [file ADVS-9999-e75782-s002.zip › Figure S4.jpg]

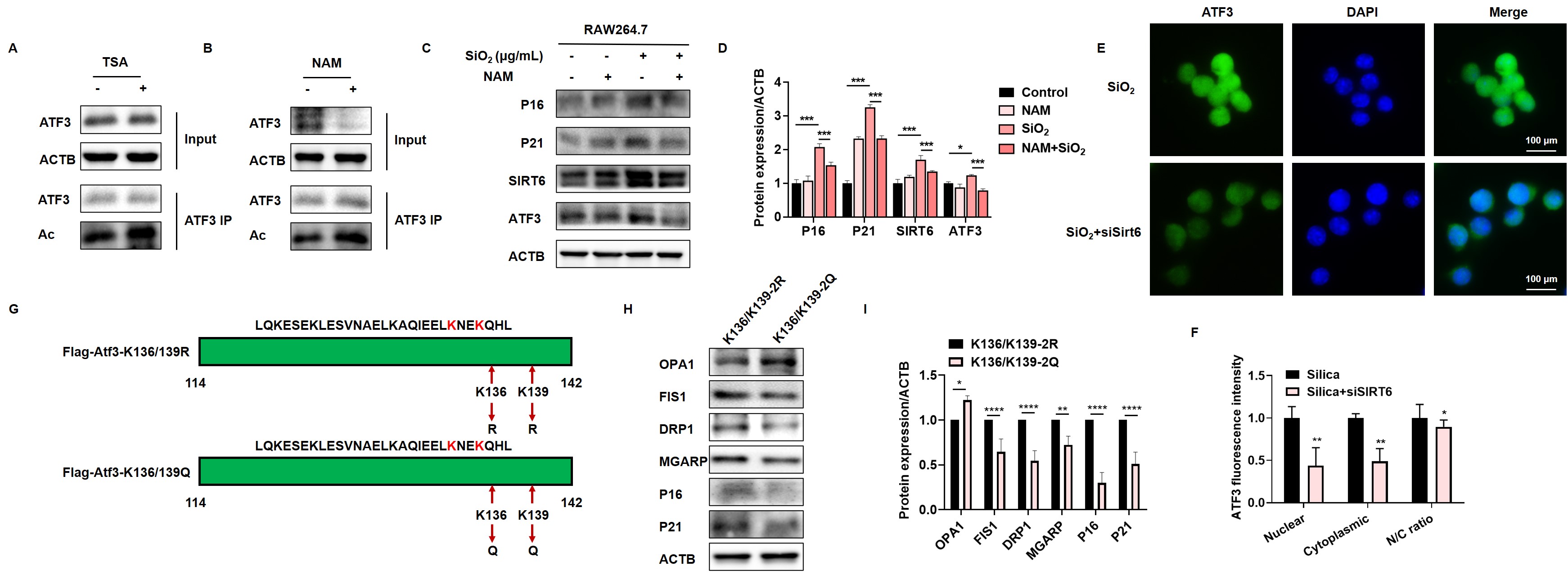

Supplement: Supplementary file 2 — Supporting File 2: advs75782‐sup‐0002‐FigureS1‐S8.zip. [file ADVS-9999-e75782-s002.zip › Figure S5.jpg]

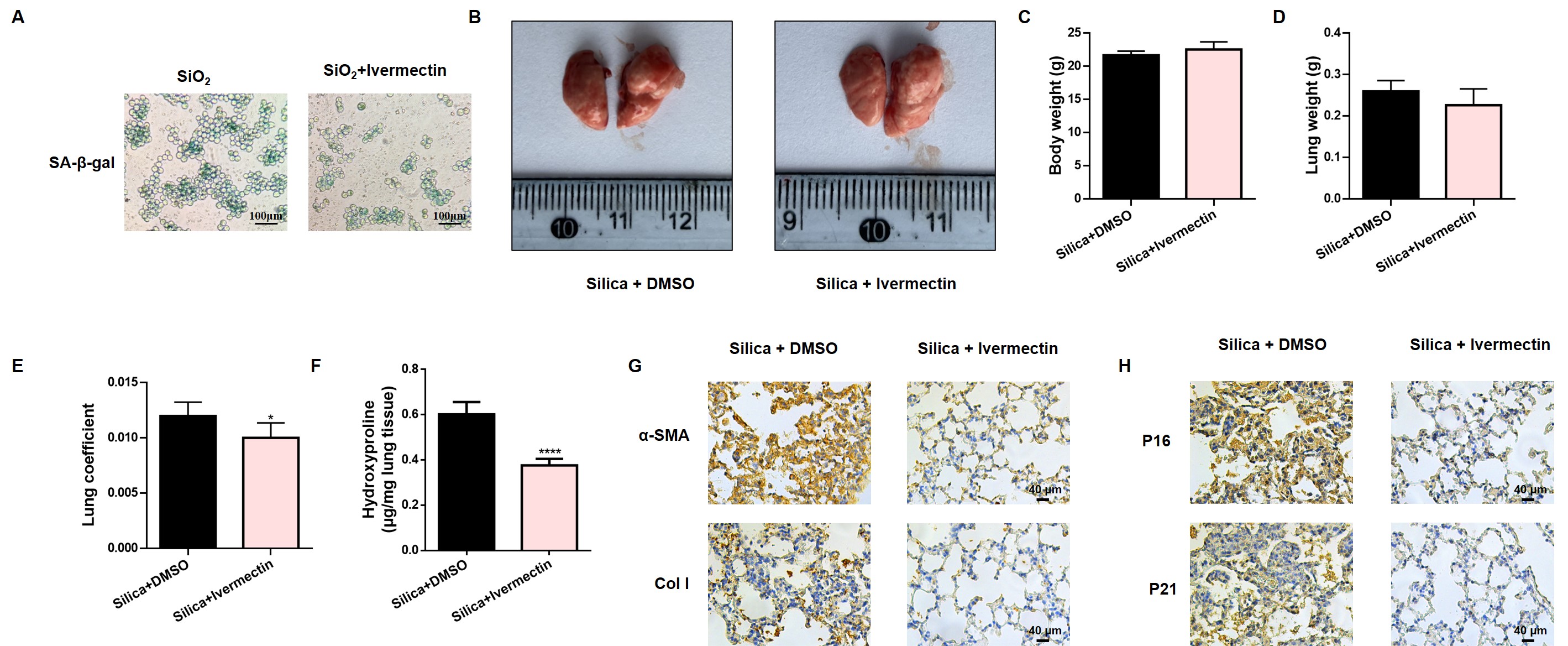

Supplement: Supplementary file 2 — Supporting File 2: advs75782‐sup‐0002‐FigureS1‐S8.zip. [file ADVS-9999-e75782-s002.zip › Figure S6.jpg]

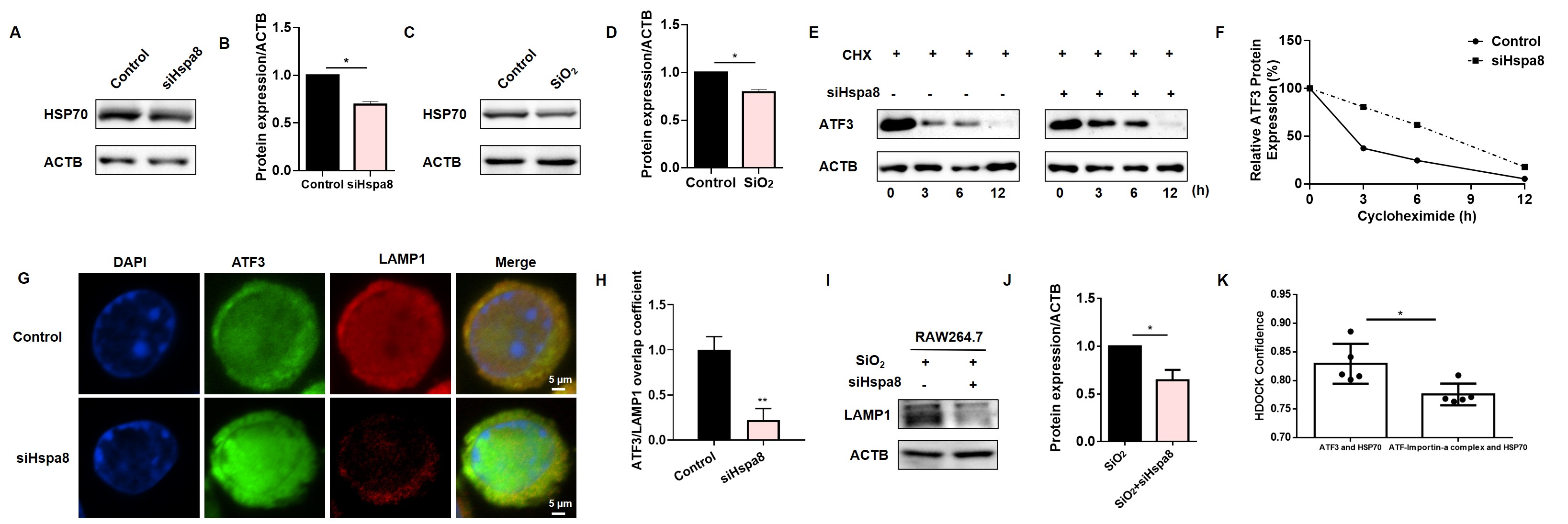

Supplement: Supplementary file 2 — Supporting File 2: advs75782‐sup‐0002‐FigureS1‐S8.zip. [file ADVS-9999-e75782-s002.zip › Figure S7.jpg]

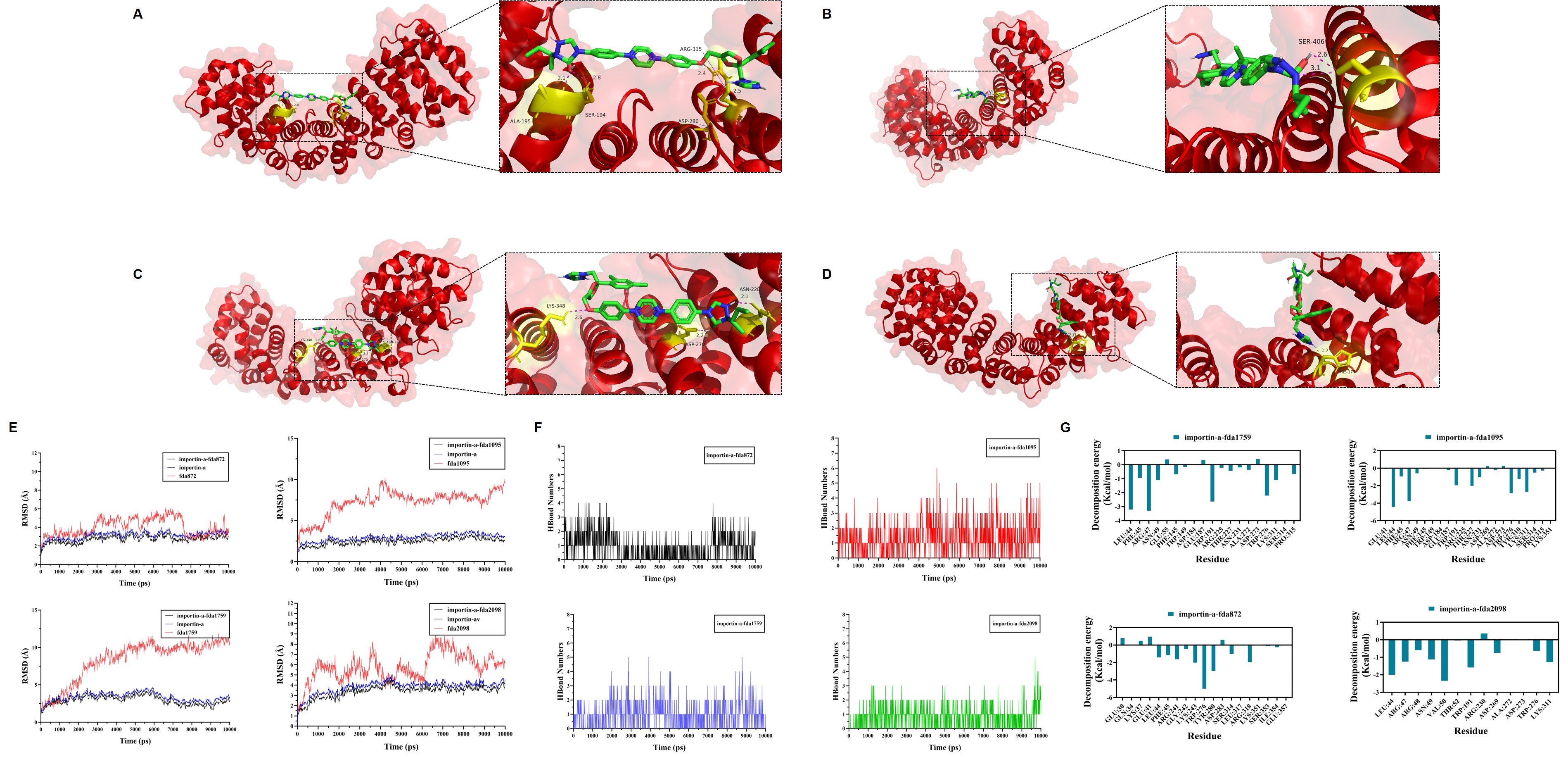

Supplement: Supplementary file 2 — Supporting File 2: advs75782‐sup‐0002‐FigureS1‐S8.zip. [file ADVS-9999-e75782-s002.zip › Figure S8.jpg]
